# Tamm-Horsfall protein augments neutrophil NETosis during urinary tract infection

**DOI:** 10.1172/jci.insight.180024

**Published:** 2025-01-09

**Authors:** Vicki Mercado-Evans, Holly Branthoover, Claude Chew, Camille Serchejian, Alexander B. Saltzman, Marlyd E. Mejia, Jacob J. Zulk, Ingrid Cornax, Victor Nizet, Kathryn A. Patras

**Affiliations:** 1 Department of Molecular Virology and Microbiology,; 2 Medical Scientist Training Program,; 3 Cytometry and Cell Sorting Core, and; 4 Mass Spectrometry Proteomics Core, Baylor College of Medicine (BCM), Houston, Texas, USA.; 5 Department of Pediatrics and; 6 Skaggs School of Pharmacy and Pharmaceutical Sciences, UCSD, La Jolla, California, USA.; 7 Alkek Center for Metagenomics and Microbiome Research, BCM, Houston, Texas, USA.

**Keywords:** Immunology, Infectious disease, Mouse models, Neutrophils, UTI/pyelonephritis

## Abstract

Urinary neutrophils are a hallmark of urinary tract infection (UTI), yet the mechanisms governing their activation, function, and efficacy in controlling infection remain incompletely understood. Tamm-Horsfall glycoprotein (THP), the most abundant protein in urine, uses terminal sialic acids to bind an inhibitory receptor and dampen neutrophil inflammatory responses. We hypothesized that neutrophil modulation is an integral part of THP-mediated host protection. In a UTI model, THP-deficient mice showed elevated urinary tract bacterial burdens, increased neutrophil recruitment, and more severe tissue histopathological changes compared with WT mice. Furthermore, THP-deficient mice displayed impaired urinary NETosis during UTI. To investigate the effect of THP on NETosis, we coupled in vitro fluorescence-based NET assays, proteomic analyses, and standard and imaging flow cytometry with peripheral human neutrophils. We found that THP increases proteins involved in respiratory chain, neutrophil granules, and chromatin remodeling pathways; enhances NETosis in an ROS-dependent manner; and drives NET-associated morphologic features including nuclear decondensation. These effects were observed only in the presence of a NETosis stimulus and could not be solely replicated with equivalent levels of sialic acid alone. We conclude that THP is a critical regulator of NETosis in the urinary tract, playing a key role in host defense against UTI.

## Abstract 2

This study finds that a urinary protein enhances neutrophil NETosis, an important antimicrobial response, during urinary tract infection.

## Introduction

Urinary tract infections (UTI) affect 400 million people globally each year, with about 50% of women experiencing at least 1 UTI in their lifetime (1). The most common UTI culprit, responsible for 75% of cases, is uropathogenic *E. coli* (UPEC) (2). Genetic factors increasing UTI susceptibility include variants in pathogen recognition, innate immunity, and neutrophil recruitment (3). Rapid recruitment of neutrophils is a hallmark feature of UTI (4, 5) corresponding with the onset of UTI symptoms (6). Neutrophils, the initial responders to UTI, are detected in urine as early as 2 hours after infection, and appropriate recruitment is required for UTI resolution in murine models (7, 8). Aberrant neutrophil recruitment in mice leads to pathological neutrophil accumulation, tissue damage, and scarring (9), whereas neutrophil depletion exacerbates bacterial burdens and promotes chronic infection (7, 10).

Neutrophils display diverse antibacterial functions contributing to UTI resolution. They are a critical source of antimicrobial proteins including cathelicidin (11, 12) and lactoferrin (13) and are the principal cells performing bacterial phagocytosis in vivo (14). Additionally, neutrophils are a key source of reactive oxygen species (ROS), essential for bacterial killing, but which in excess can promote tissue damage, particularly in the kidneys (15). Neutrophils from patients with recurrent UTI display decreased phagocytosis and reduced production of reactive oxygen intermediates, underscoring the importance of these functions for neutrophil antibacterial activity and UTI resolution (16).

Another neutrophil antimicrobial mechanism is NETosis — the process of forming neutrophil extracellular traps (NETs) (17). NETosis is a form of cell death resulting in expulsion of a decondensed chromatin scaffold studded with antimicrobial products, such as myeloperoxidase (MPO), cathelicidin, and histones, that trap extracellular pathogens aiding in infection control (17, 18). Multiple stimuli can trigger NETosis, including phorbol-myristate acetate (PMA), LPS, calcium ionophores, hydrogen peroxide, and various microbes, such as fungi and Gram-positive and Gram-negative bacteria (19). Distinct subtypes of NETosis, discriminated based on cellular morphology and signaling pathways, include classical (or suicidal) NETosis; mitochondrial NETosis, where NETs are formed from mitochondrial DNA; and nonclassical (or vital) NETosis, where the neutrophil expels nuclear DNA without or prior to lysing (20–22). While recent studies have reported the presence of NET-associated products (e.g., DNA, histones, MPO) in the urine of patients with UTI (23) and have demonstrated NET formation in a UTI bladder-on-a-chip model (24), the role of NETosis in UTI susceptibility and resolution remains to be established.

We hypothesized that urinary factors may influence NET formation in UTI. Tamm-Horsfall protein (THP, or uromodulin), the most abundant urinary protein, is a highly conserved glycoprotein with important roles in urinary tract health, including regulation of salt and water homeostasis and prevention of mineral crystallization (25). In the context of UTI, THP is a key host defense factor. Elimination of THP increases UTI susceptibility in murine models (26–29). THP directly binds pathogens (29–31), inhibiting adherence to host urothelium, which in turn aids clearance via urinary excretion. THP also shapes host responses to UPEC by modulating immune cell activity in a cell type– and context-dependent manner (32, 33). We previously showed that THP terminal sialic acids engage Siglec-9, an inhibitory receptor, to suppress neutrophil activities including chemotaxis, ROS release, and bactericidal capacity (34). This immunosuppressive effect of THP is revealed in THP-KO mice, which exhibit elevated proinflammatory cytokines, increased kidney inflammation upon injury, and neutrophilia in the blood and urine with or without inflammatory stimuli (34–36).

Given the protective roles of THP and neutrophils in UTI, and considering THP influence on neutrophil responses, we hypothesized that THP may provide additional host protection by modulating NETosis. To investigate this hypothesis, we evaluated neutrophil recruitment and NETosis in a UTI model comparing WT to THP-deficient mice. Our findings reveal increased bladder neutrophils in THP-deficient mice but reveal reduced NET formation compared with WT mice. Subsequent validation through flow cytometry of human neutrophils confirmed that THP enhancement of NETosis was dependent on neutrophil activation and ROS and did not mimic caspase-mediated forms of cell death. In conjunction with its roles in impeding pathogen adherence and tempering excessive inflammation, we conclude that THP provides added host protection by modulating NETosis during UTI.

## Results

### THP deficiency increases urinary tract UPEC burdens and tissue histopathology.

**Figure 1:**
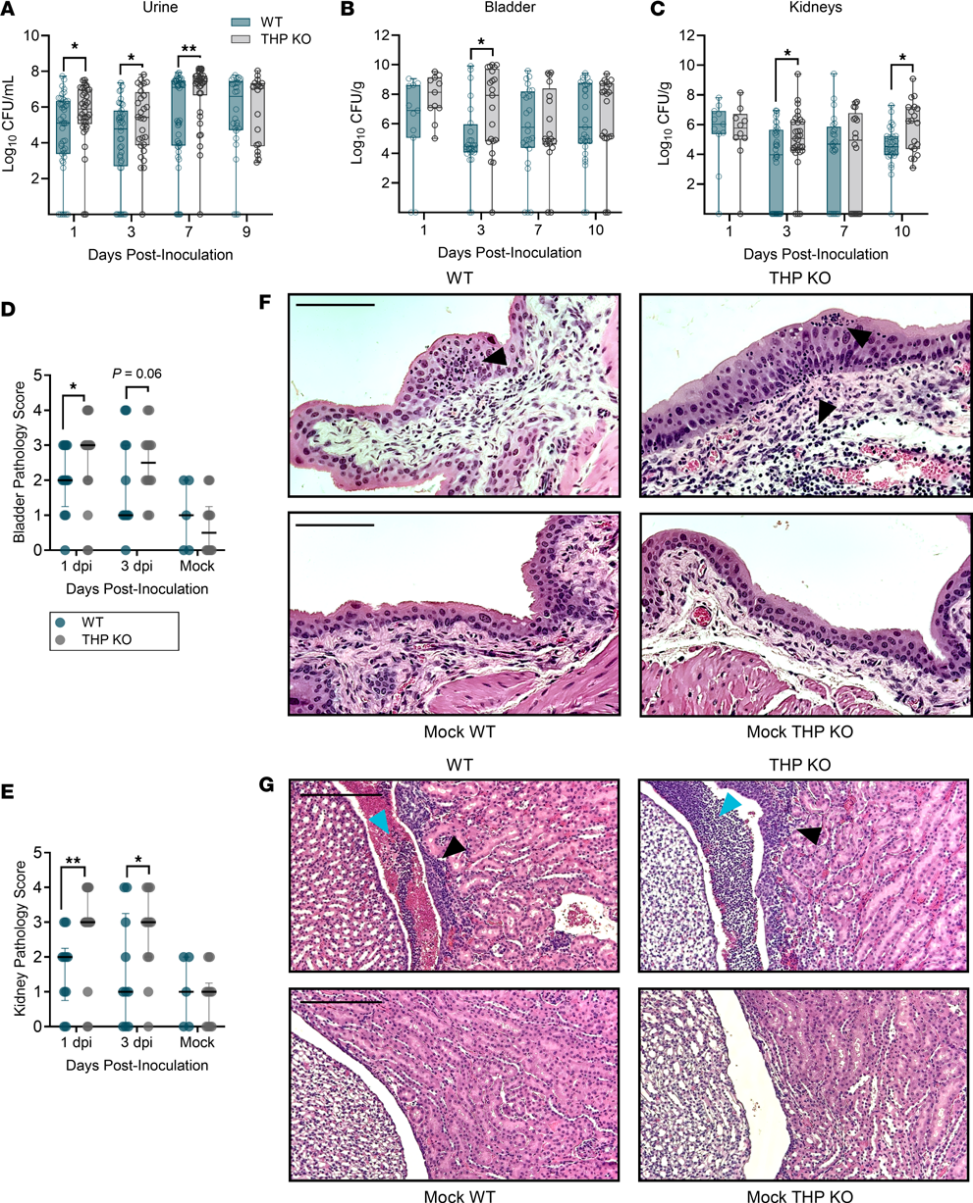
THP deficiency increases urinary tract bacterial burdens and tissue pathology. (**A**–**C**) Time course of urine (**A**), bladder (**B**), and kidney (**C**) UPEC burdens from WT and THP-KO mice. Urine samples were collected from mice at multiple time points up until tissue collection. (**D** and **E**) Bladder (**D**) and kidney (**E**) pathology scores on 1 and 3 dpi. (**F** and **G**) Representative H&E images of day 1 bladders (**F**) and day 3 kidneys (**G**) from UPEC-infected or mock-infected mice. Scale bars: 110 μm (**F**) and 210 μm (**G**). Arrowheads point to polymorphonuclear cell infiltration (black) and polymorphonuclear cell aggregates (blue). Experiments were performed at least twice with data combined. *n* = 18–46/time point (**A**), *n* = 11–31 (**B** and **C**), or *n* = 5–15 (**D** and **E**). Box-and-whisker plots show median, all points, and 25–75th percentiles (**A**–**C**). Points represent individual samples; lines indicate medians with interquartile ranges (**D** and **E**). Data were analyzed by Mann-Whitney *U* test (**A**–**C**) and Fisher’s exact test (**D** and **E**). *P < 0.05; **P < 0.01.

Prior studies have identified the heightened UTI susceptibility of THP-deficient mice at 24 hours after infection (26, 27). To assess the sustained effect of THP deficiency, we used an established model of UTI with cystitis strain UTI89 (37) in THP^+/+^ (WT) and THP^–/–^ (KO) mice (34). Consistent with previous findings (26, 27), THP-KO mice exhibited persistent increased bacteriuria (Figure 1A) and temporarily elevated bacterial tissue loads (Figure 1, B and C) compared with WT mice throughout the infection course. Bladder and kidney sections were examined by a blinded veterinary pathologist and scored on a 0–4 scale, considering pathologic features such as intraluminal bacteria, submucosal edema, and suppurative (pus-forming) pyelonephritis. UPEC-infected THP-KO mice displayed more severe tissue pathology compared with WT counterparts (Figure 1, D and E). Infected bladders were marked by increased immune infiltration in the urinary epithelium and submucosa (Figure 1F). Suppurative pyelonephritis was the most common renal histopathology and, when evident, tended to occur within, or adjacent to, the renal pelvis (Figure 1G). Hydronephrosis and lymphohistiocytic pyelonephritis were occasionally observed. No differences in tissue histopathology were observed between mock-infected WT and THP-KO groups.

### THP deficiency alters bladder neutrophil infiltration and the effect of neutrophil depletion during UTI.

**Figure 2:**
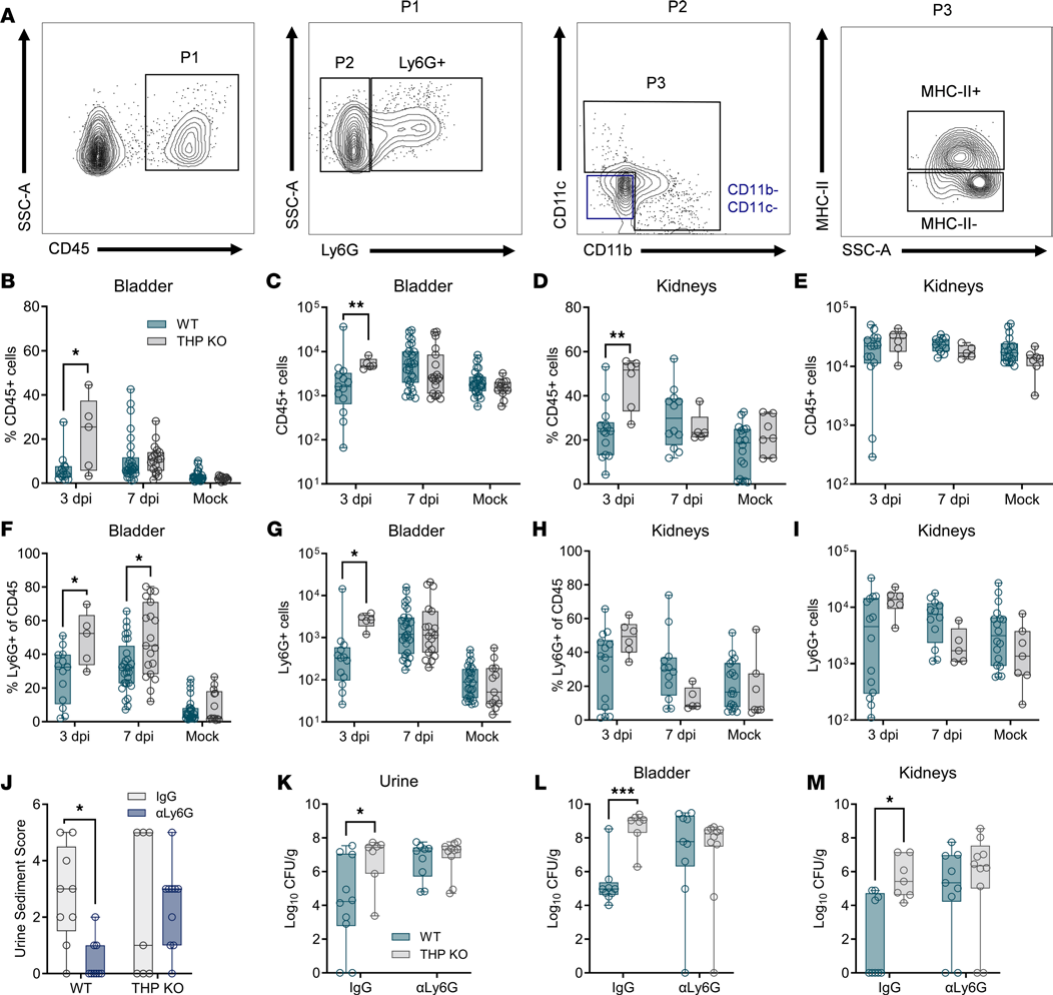
THP deficiency increases bladder neutrophil infiltration and reduces bacterial burdens upon neutrophil depletion. (**A**) Gating strategy for quantifying immune populations of interest with a focus on neutrophils (Ly6G^+^) and myeloid lineages (CD11b^+/–^, CD11c^+/–^). (**B**–**E**) Frequency and counts of CD45^+^ cells (P1) cells in bladder or kidneys at 3 and 7 dpi. Mock-infected samples from both time points were combined prior to analyses. (**F**–**I**) Frequency and counts of neutrophils (Ly6G^+^) from CD45^+^ populations infiltrating bladder or kidneys. Mice were administered anti-Ly6G or IgG isotype control prior to bacterial inoculation and on 2 and 4 dpi. (**J**) Urine sediment scores at 6 dpi. (**K**) Urine UPEC burdens at 6 dpi. (**L** and **M**) Bladder and kidney UPEC burdens at 7 dpi. Experiments were performed at least twice and combined. *n* = 5–32/group (**B**–**I**) and *n* = 7–10 (**J**–**M**). Box-and-whisker plots show median, all points, and 25–75th percentiles (**B**–**I**). Data were analyzed by Mann-Whitney *U* test (**B**–**I**, and **K**–**M**) and Fisher’s exact test (**J**). *P < 0.05; **P < 0.01; ***P < 0.001.

We evaluated bladder and kidney immune cell infiltration by flow cytometry, surveying the total immune cell fraction (CD45^+^ [P1]), as well as neutrophils (Ly6G^+^), nonmyeloid (CD11b^–^CD11c^–^), myeloid (CD11b^+/–^CD11c^+/–^ [P3]), myeloid antigen presenting cells (APCs; MHC-II), and myeloid non-APC subpopulations (gating scheme in Figure 2A). At 3 days postinfection (dpi), THP-KO mice had higher proportions and counts of CD45^+^ cells compared with WT-infected mice, although counts in the kidneys did not reach significance (*P* = 0.076). No differences were observed in later time points or mock controls (Figure 2, B–E). Bladder neutrophil proportions and counts were elevated in infected THP-KO mice compared with WT mice at 3 dpi, with no observed differences in kidneys or mock controls (Figure 2, F–I). At 7 dpi, THP-KO bladders showed depressed frequency and counts of myeloid cells with decreased myeloid non-APC and myeloid APC numbers (Supplemental Figure 1; supplemental material available online with this article; https://doi.org/10.1172/jci.insight.180024DS1). Other bladder immune cell subpopulations did not differ between groups. In the kidneys, minimal differences in other subpopulations were noted, including an increased proportion and number of lymphocytes and reduced proportion of myeloid lineages at 3 dpi (Supplemental Figure 2). Under mock-infected conditions, THP-KO mice exhibited a slight but significant decrease in lymphocyte and APC counts and an increased proportion of non-APC myeloid cells (Supplemental Figure 2).

Neutrophil depletion exacerbates bacterial burdens and promotes chronic infection depending on the extent of neutrophil reduction (10). To evaluate the effect of neutrophil depletion in THP-deficient mice, we administered anti-Ly6G antibody or isotype IgG i.p. every 48 hours, from 0 to 6 dpi. On 6 dpi, urine sediment was scored for the presence of polymorphonuclear (PMN) cells on a scale of 0–5 as described previously (10). Urine and tissues were collected on 7 dpi to quantify bacterial burden. Anti-Ly6G antibody treatment significantly reduced urine sediment PMN scores in WT mice but had no such effect in THP-KO mice (Figure 2J). Additionally, anti-Ly6G antibody treatment resolved bacterial urine and tissue burdens between WT and THP-KO mice, highlighting the importance of neutrophils in mediating increased susceptibility (Figure 2, K–M). We next probed the crosstalk between cyclooxygenase 2 (COX-2), a critical enzyme that initiates inflammatory cascades, and THP-mediated UTI susceptibility. COX-2 downregulates THP kidney expression, and COX-2^–/–^ mice are hypersusceptible to UTI (38), thus we examined the effect of COX-2 in our model. Mice were treated with diclofenac, a COX-2 inhibitor (39), in drinking water from 0 to 6 dpi, with tissues collected at 7 dpi. We found no differences in tissue burdens between diclofenac-treated and mock-treated WT and THP-KO mice (Supplemental Figure 3), suggesting that enhanced UTI susceptibility in THP-KO mice is independent of COX-2 inflammatory pathways in this model. Together, these findings highlight that elevated neutrophils are a distinctive feature of THP deficiency in UTI, which, paired with enhanced bacterial burdens, suggest impaired neutrophil activity in THP-KO mice.

### Murine urinary THP levels and glycosylation change minimally during UTI.

**Figure 3:**
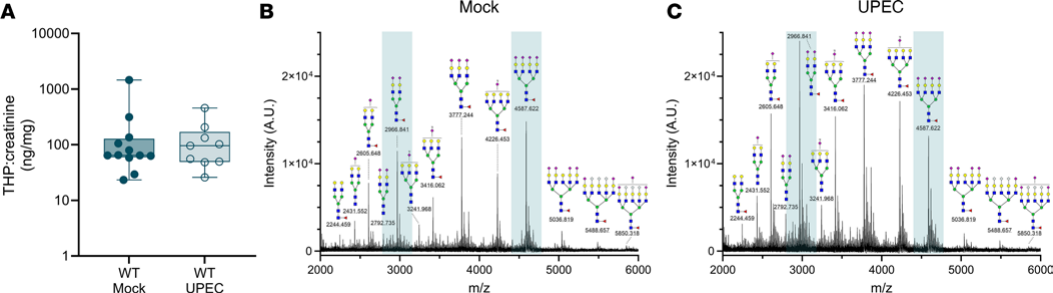
Tamm-Horsfall protein levels and glycosylation patterns change minimally during urinary tract infection in vivo. (**A**) THP urine levels normalized to creatinine in mock-infected (or baseline day 0) and infected mice (1–4 dpi combined). Box-and-whisker plots show median, all points, and 25-75th percentiles (*n* = 9–12). Data were collected from 3 experiments. N-glycan MALDI-TOF profiles of THP isolated from WT mice that were either mock- (**B**) or UPEC-infected (**C**). Data represent 1 analysis of purified THP harvested from pooled urine from WT mice (*n* = 15 mock, *n* = 28 UPEC) collected as a part of 2 independent experiments. Prominent peaks with proportional differences between UPEC-infected and mock samples (*m/z* 2,967 and 4,588) are highlighted in teal. Data in **A** were analyzed by Mann-Whitney *U* test.

Clinical studies have linked *UMOD* variants (40) and reduced THP production (41, 42) with enhanced UTI risk, although a cross-sectional study found no differences in urinary THP levels between pediatric patients with UTI and controls (43). Similarly, we observed no variations in urinary THP levels between mock-infected and UPEC-infected WT mice (Figure 3A). To delineate the N-glycan profile of murine THP and assess the effect of UTI on THP glycosylation, we collected urine over 96 hours after inoculation and profiled THP glycosylation patterns using Matrix-Assisted Laser Desorption/Ionization TOF/TOF mass spectrometry (MALDI-TOF/TOF MS). Similar to human THP (44–46), murine THP contained multiple bi-, tri-, and tetraantennary sialylated and/or fucosylated complex type N-glycans (Figure 3B). The highest intensity peak (*m/z* 4,588) represented a tetraantennary, tetrasialylated and fucosylated N-glycan (Figure 3B and Supplemental Table 1), which matches the most abundant N-glycan on human THP (44, 45). Other high-intensity peaks were observed at *m/z* 2,967; 3,777; and 4,226. In UPEC-infected THP samples, these 4 most abundant structures remained the same, albeit with some proportional differences: the *m/z* 2,967 peak increased and the *m/z* 4,588 peak decreased relative to mock-treated spectra (Figure 3C and Supplemental Table 1). We quantified total sialic acids released from murine THP by abeling sialic acids with 1,2-diamino4,5-methylenedioxybenzene (DMB) and measuring via high-performance liquid chromatography (HPLC) with fluorescence detection. No differences in N-glycolylneuraminic acid (Neu5Gc), N-acetylneuraminic acid (Neu5Ac), or total sialic acid levels were observed between mock-infected and UPEC-infected samples (Table 1). Samples from THP-KO mice showed significantly reduced Neu5Ac and total sialic acid compared with WT mock samples, validating that THP was the primary source of sialic acid (Table 1). Together, these data reveal conserved glycosylation patterns, including sialylation by Neu5Ac, in murine and human THP that are retained during murine UTI.

### Murine neutrophils undergo NETosis during UTI, and THP deficiency alters neutrophil subpopulations.

**Figure 4:**
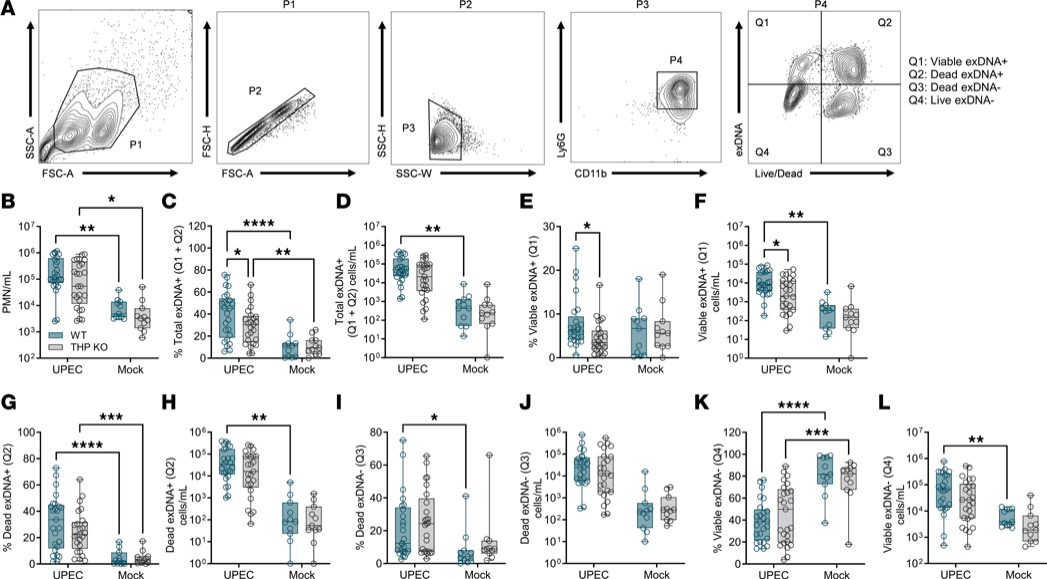
Neutrophil exDNA^+^ populations are decreased in THP-deficient mice during UTI. (**A**) Gating strategy for quantifying neutrophil (PMNs, Ly6G^+^CD11b^+^ [P4]) subpopulations of interest including viable exDNA^+^ (extracellular DNA[SYTOX Orange]^+^Live/Dead^–^), dead exDNA^+^ (exDNA^+^Live/Dead^+^), dead PMNs (exDNA^–^Live/Dead^+^), and live PMNs (exDNA^–^Live/Dead^–^). (**B**) Total PMNs (P4) per mL of urine. (**C** and **D**) Frequency out of total PMNs and counts of total exDNA^+^ cells (Q1+Q2). (**E** and **F**) Frequency of and counts of viable exDNA^+^ cells. (**G** and **H**) Frequency of and counts of dead exDNA^+^ cells. (**I** and **J**) Frequency of and counts of dead exDNA^–^ cells. (**K** and **L**) Frequency of and counts of live exDNA^–^ cells. Experiments were performed at least twice and combined. *n* = 11–15/group. Box-and-whisker plots show median, all points, and 25–75th percentiles. Data were analyzed by 2-way ANOVA with uncorrected Fisher’s LSD test. *P < 0.05; **P < 0.01; ***P < 0.001; ****P < 0.0001.

To investigate whether differences in neutrophil abundance corresponded with differences in neutrophil function, we characterized NETosis using several methods. Nucleic acid dyes (e.g., Hoechst) and non–cell permeable SYTOX dyes have distinguished NETosis from other forms of cell death in mixed-cell populations (47–49). Additionally, plasma membrane permeability can be confirmed using a live/dead amine-reactive dye that only labels intracellular amines if the membrane is compromised (50). In classical NETosis, neutrophils permeabilize and expel decondensed chromatin, whereas during nonclassical NETosis, neutrophils release DNA but retain viability and effector functions (51, 52). We subjected mouse urine collected 24 hours after inoculation to flow cytometry. Neutrophils (PMNs) were identified as CD11b^+^Ly6G^+^ and were further gated based on presence of extracellular DNA (exDNA; SYTOX Orange [SO)] and plasma membrane permeability (Live/Dead stain) as depicted in Figure 4A. We identified 4 unique populations: viable PMNs (SO^–^Live/Dead^–^ [Q4]), dead PMNs (SO^–^Live/Dead^+^ [Q3]), dead PMNs with exDNA (SO^+^Live/Dead^+^ [Q2]), and viable PMNs with exDNA (SO^+^Live/Dead^–^ [Q1]). WT and THP-KO mice displayed increased urinary neutrophils during infection compared with mock-infected counter parts (Figure 4B). UPEC infection elevated proportions and total counts of exDNA cells (and dead and viable subsets), most notably in WT mice, compared with their mock-infected counterparts (Figure 4, C–H). Uniquely, WT mice showed elevated frequency and counts of viable PMNs with exDNA in response to UPEC infection and compared with UPEC-infected THP-KO mice (Figure 4, E and F). Dead PMNs were observed at a higher frequency in WT mice in response to infection (Figure 4, I and J). In both WT and THP-KO mice, frequency of live PMNs was reduced during infection, but WT sustained higher total live PMN numbers during infection compared with mock-infected WT mice (Figure 4, K and L).

**Figure 5:**
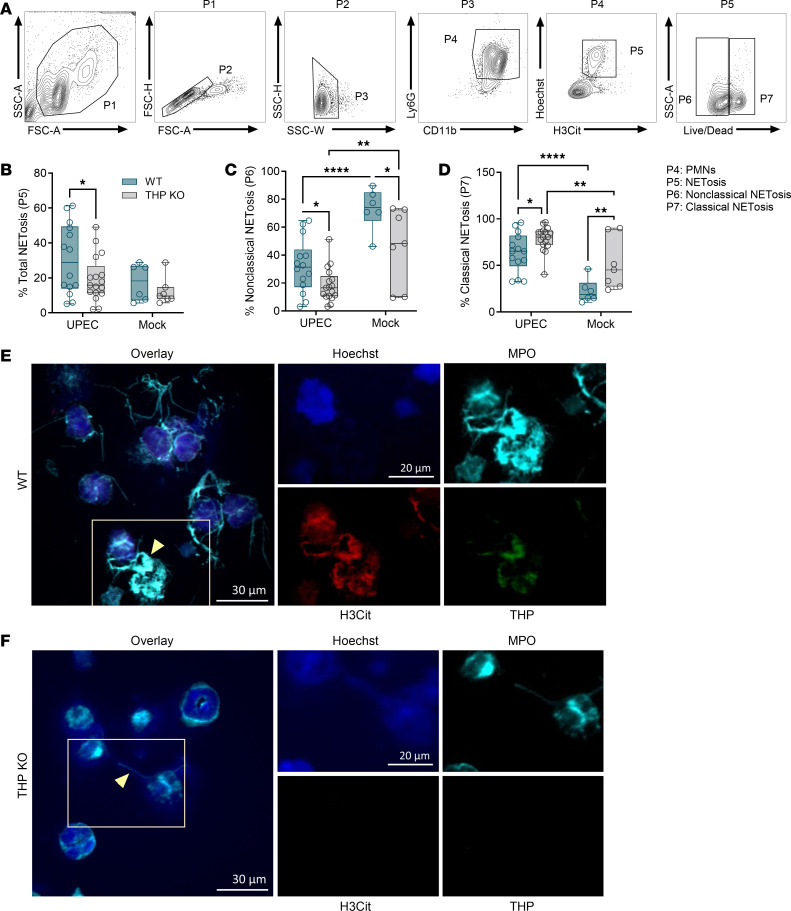
Neutrophil NETosis populations are altered in THP-deficient mice during UTI. (**A**) Gating strategy for quantifying neutrophil (PMNs, Ly6G^+^CD11b^+^ [P4]) subpopulations of interest with a focus on NETosis (H3Cit^+^Hoechst^+^), and nonclassical (H3Cit^+^Live/Dead^–^) and classical (H3Cit^+^Live/Dead^+^) subsets. (**B**) Frequency of NETosis in total PMNs. (**C** and **D**) Frequency of nonclassical and classical subsets in total NETosis population. (**E** and **F**) Urine samples from UPEC-infected WT and THP-KO mice were mounted on slides and NETs were visualized via immunofluorescence using antibodies against myeloperoxidase (MPO, cyan), citrullinated histone H3 (H3Cit, red), and THP (green). Nucleic acids were stained using Hoechst dye (blue). Yellow arrowheads point to NET structures depicted as strands of DNA dotted with MPO staining. Experiments were performed at least twice and combined. *n* = 6–17/group. Box-and-whisker plots show median, all points, and 25–75th percentiles. Data were analyzed by 2-way ANOVA with uncorrected Fisher’s LSD test. *P < 0.05; **P < 0.01; ****P < 0.0001.

To confirm the presence of NETs, we assessed citrullinated modifications on Histone 3 (citrullinated histone H3 [H3Cit]), catalyzed by the enzyme peptidylarginine deiminase 4 (PAD4), which contributes to chromatin decondensation and NET formation (53). Neutrophils (PMNs) were identified as CD11b^+^Ly6G^+^ and were further gated based on staining for presence of H3Cit and plasma membrane permeability (Live/Dead stain) as depicted in Figure 5A. During infection, WT neutrophils displayed higher frequency of NETosis compared with THP-KO mice (Figure 5B). When separated into nonclassical NETosis and classical NETosis subsets based on membrane permeability, WT neutrophils showed greater proportions of nonclassical NETosis and decreased classical NETosis compared with THP KO in both infected and mock-infected states (Figure 5, C and D). The presence of NETs in WT and THP-KO urine samples was visualized by immunofluorescence microscopy using antibodies for neutrophils (MPO), NETosis (H3Cit), and THP (Figure 5, E and F).

### THP enhances NETosis in human neutrophils with minimal effects on cellular proteins.

**Figure 6:**
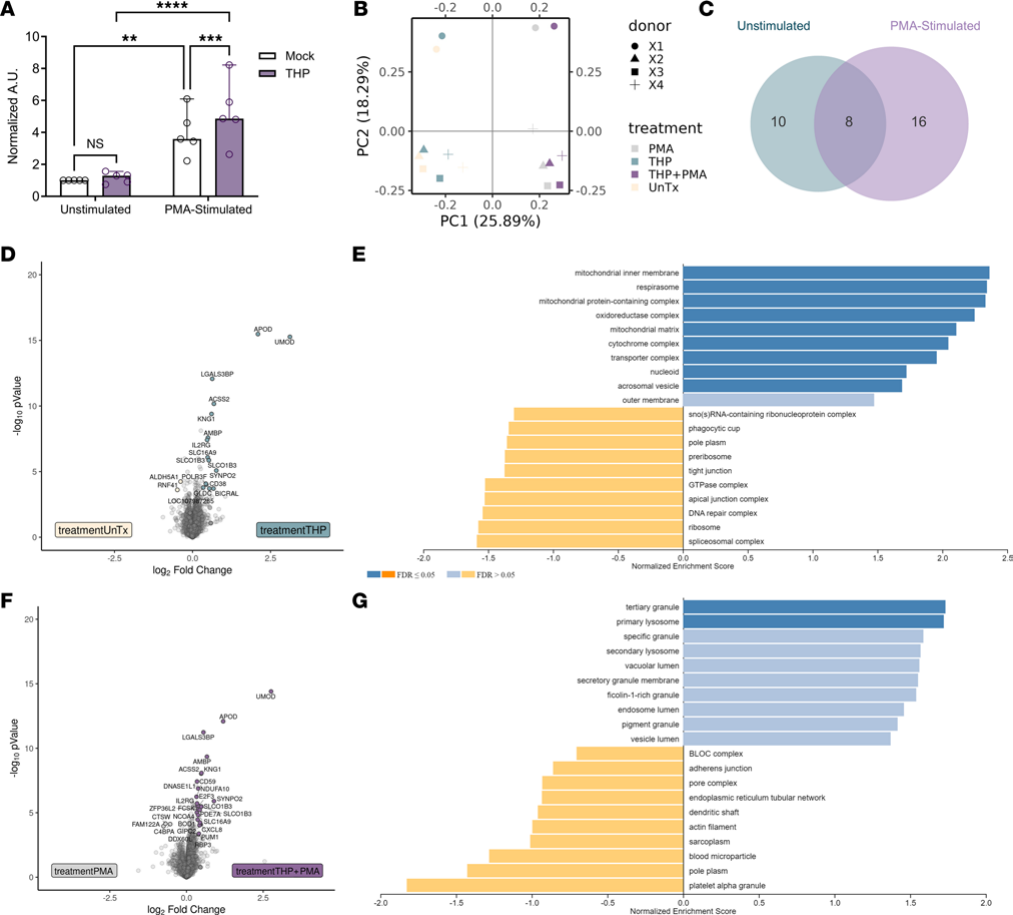
THP modestly alters neutrophil responses to PMA stimulation. (**A**) NETosis was assessed by released dsDNA (detected by PicoGreen dye) and expressed as arbitrary units (AU) of fluorescence normalized to mock-treated, unstimulated controls. Neutrophils were subjected to tandem mass tag-based proteomics profiling. (**B**) Principal component analysis of neutrophils that were untreated (UnTx), treated with THP (THP), untreated with PMA stimulation (PMA), and THP-treated with PMA stimulation (THP+PMA). Points represent individual samples, colored by treatment, with paired donor samples indicated by matched symbol. (**C**) Venn diagram of proteins differentially detected in THP-treated samples compared with untreated samples in PMA-stimulated (PMA and THP+PMA) and unstimulated (UnTx and THP) conditions. (**D** and **E**) Volcano plot (**D**) and gene set enrichment analysis (**E**) of differential proteins in untreated versus THP-treated samples. (**F** and **G**) Volcano plot (**F**) and gene set enrichment analysis (**G**) of differential proteins in PMA versus THP+PMA samples. Experiments were performed independently 3 times and combined, *n* = 5 (**A**), or as one independent experiment, *n* = 4 (**B**–**G**). Bar plots show median, all points, and 95% CI (**A**). Data were analyzed by 2-way ANOVA with Šídák’s multiple-comparisons test (**A**). Differential proteins (**C**–**G**) were identified via log_2_ fold change > 1.25 and moderated 2-tailed *t* test followed by multiple-hypothesis testing correction using the Benjamini-Hochberg procedure with a FDR-adjusted *P* < 0.05. Individual proteins are listed in Supplemental Table 2.

To determine if THP’s effect on NETosis extended to human models, we measured NETosis formation in human neutrophils with and without THP exposure at physiologic concentrations. After 2.5 hours of PMA stimulation, NETosis was measured by detection of fluorescently labeled exDNA as described previously (13). THP pretreatment increased exDNA in PMA-stimulated cells but not in unstimulated cells (Figure 6A). To identify cellular processes affected by THP, we performed quantitative proteomics of neutrophils under these same 4 conditions: mock-treated unstimulated (UnTx), THP-treated, PMA-stimulated, and PMA-stimulated and THP-treated (PMA+THP). PMA stimulation was the primary driver of variation between samples as shown by PCA (Figure 6B) and resulted in depleted neutrophil granule and NETs-related proteins likely due to the release of these proteins from activated cells (Supplemental Figure 4). Eight shared proteins were increased in THP and PMA+THP samples compared with their mock-treated counterparts (Figure 6C and Supplemental Tables 2 and 3). These proteins included THP itself (UMOD) and other known urinary proteins: apolipoprotein D, alpha-1-Microglobulin/Bikunin Precursor (AMBP), kininogen (KNG1), and galectin 3 binding protein (LGALS3BP) (54). MS analysis of purified THP confirmed presence of THP (77.43%). Several other proteins were detected at > 0.5%: apolipoprotein D, E3 ubiquitin-protein ligase (MIB2), protein AMBP, Metabolism Of Cobalamin Associated D (MMADHC), LGALS3BP, β-actin (ACTB), and KNG1 (Supplemental Table 4). The remaining 3 shared proteins, not detected in purified THP, were related to cellular metabolism (ACSS2, SLC16A9) and immune signaling (IL-2R-gamma). In unstimulated cells, 10 additional proteins were differentially abundant between THP-treated and mock-treated conditions (Figure 6D and Supplemental Table 2) and included several related to translational regulation and protein turnover (EIF2AK4, POLR3F, UBAC1), second messenger signaling (CD38), cytokine receptor signaling (RNF41), mitochondrial metabolism (GLDC, ALDH5A1), phagosome acidification and fusion (RAB20), and chromatin remodeling (BICRAL). Gene ontology (excluding proteins detected in purified THP) identified mitochondrial respiratory chain complexes as significantly enriched in THP-treated conditions (Figure 6E). In PMA-stimulated cells, 16 unique proteins were differentially abundant between THP-treated and mock-treated conditions (Figure 6F and Supplemental Table 2). These included proteins involved in second messenger and cell signaling (PDE7A, FCSK), transcriptional and translational regulatory proteins (PUM1, E2F3, ZFP36L2, GTPBP6, CCDC86), complement-related and immune related proteins (CD59, CXCL8, C4BPA, CTSW), intracellular trafficking and cytoskeleton arrangement (GIPC2, NCOA4, XIRP2), and DNA/chromatin remodeling proteins (DNASE1L1, BOD1). Gene ontology analyses identified significant enrichment of tertiary granule and primary lysosome pathways in THP-treated conditions, as well as nonsignificant enrichment of specific, secretory, ficolin-1–rich and pigment granules as well as vacuolar and vesicle pathways (Figure 6G). This proteomic profiling suggests that THP induces subtle differential responses related to mitochondrial metabolism in the absence of PMA stimulation and affects multiple nuclear, organelle, and cytoskeletal functions in PMA-stimulated conditions.

### THP increases NETosis in human neutrophils in a ROS-dependent manner.

**Figure 7:**
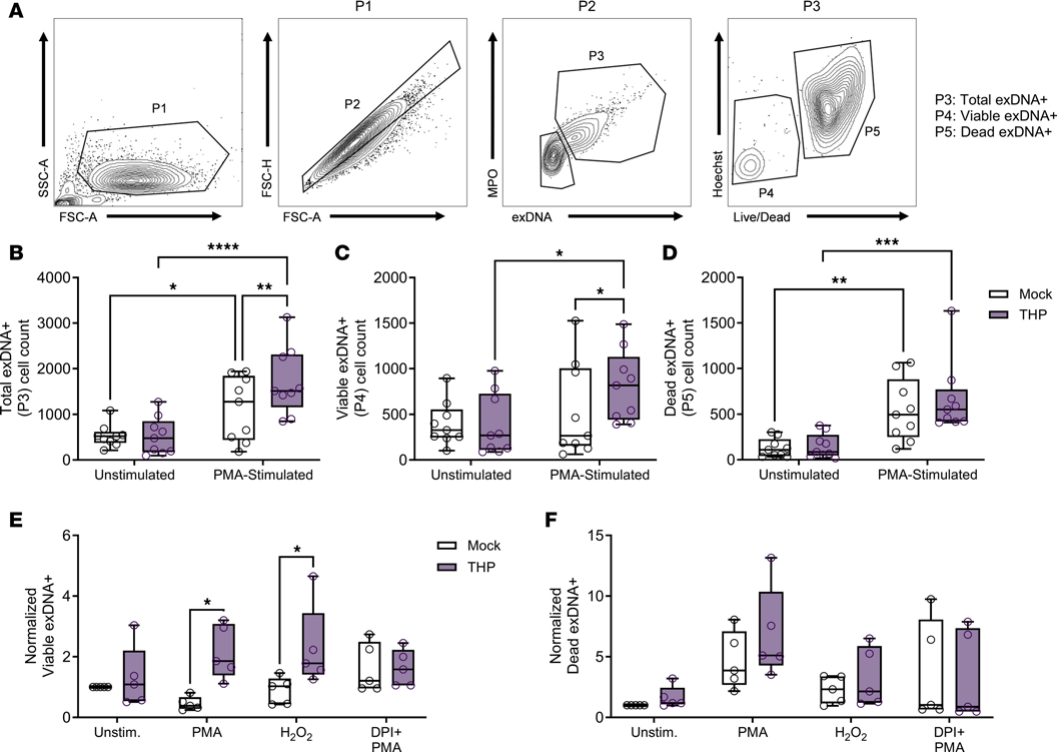
THP exposure increases viable exDNA^+^ human neutrophils. (**A**) Gating strategy for quantifying neutrophil exDNA^+^ (SYTOX Orange^+^MPO^+^ [P3]) subpopulations of interest focusing on viable (Hoechst^lo^Live/Dead^–^) and dead (Hoechst^hi^Live/Dead^+^) populations. (**B**–**D**) Cell counts for total exDNA^+^ (**B**), viable exDNA^+^ (**C**), and dead exDNA^+^ (**D**) across treatment groups. Human neutrophils were pretreated with THP or were mock treated and stimulated with either PMA, H_2_O_2_, or PMA + DPI (ROS inhibitor). (**E** and **F**) Frequency of viable exDNA^+^ cells (**E**) or dead exDNA^+^ cells (**F**) normalized to frequency of unstimulated cells from the same donor. Experiments were performed in at least 4 independent experiments and combined, *n* = 9 (**B**–**D**), or *n* = 5 (**E** and **F**). Box-and-whisker plots show median, all points, and 25–75th percentiles (**B**–**F**). Data were analyzed by 2-way ANOVA with Šídák’s multiple-comparisons test (**B**–**F**). *P < 0.05; **P < 0.01; ***P < 0.001; ****P < 0.0001.

To determine whether human neutrophils were similarly affected by THP, we modified our flow cytometry strategy for human neutrophils. Peripheral human neutrophils were treated with human THP, stimulated with PMA, and analyzed via flow cytometry. Single cells were gated for the presence of extracellular/surface neutrophil granule content (MPO) and exDNA (SO) to identify double positive cells (MPO^+^SO^+^ [P3]) (Figure 7A). P3 cells were further separated based on Hoechst and Live/Dead staining into viable exDNA^+^ (Hoechst^lo^Live/Dead^–^) and dead exDNA^+^ (Hoechst^hi^Live/Dead^+^) subsets. Consistent with the fluorescence-based NETosis assay (Figure 6A), THP significantly increased total exDNA^+^ cells in PMA-stimulated conditions but not in unstimulated cells (Figure 7B). Furthermore, THP enhanced viable exDNA^+^ (Figure 7C) and dead exDNA^+^ (Figure 7D) subsets specifically in PMA-stimulated conditions. Classical and nonclassical NETosis are dependent on NOX 2–mediated (NOX2-mediated) production of ROS (21, 55, 56), and hydrogen peroxide (H_2_O_2_), as an exogenous source of ROS, is sufficient to stimulate NETosis (19). To examine the importance of ROS on THP-mediated effects, we compared viable and dead exDNA^+^ subsets in the presence of PMA, H_2_O_2_, or PMA and a NOX inhibitor diphenyleneiodonium (DPI). THP-mediated increase in viable exDNA^+^ cells occurred in the presence of both PMA and H_2_O_2_ but was abrogated with the addition of DPI (Figure 7E). In contrast, no significant differences were observed in dead exDNA^+^ subsets under these same conditions (Figure 7F). Together, these data suggest that THP-mediated effects are in part dependent on ROS, specifically in viable exDNA^+^ cells.

**Figure 8:**
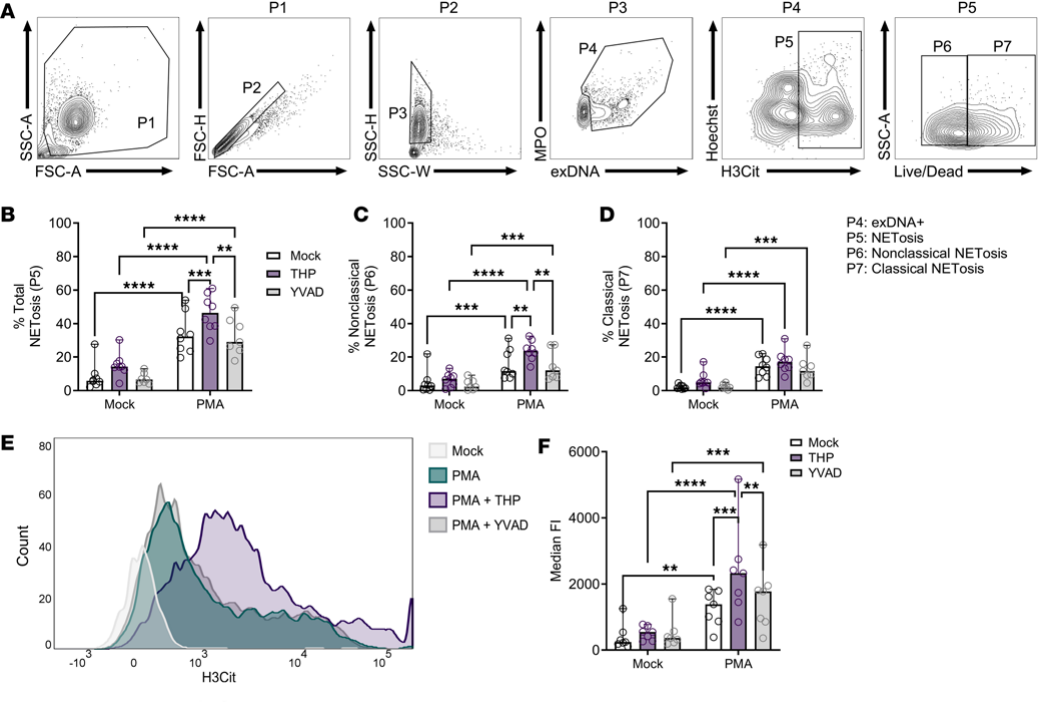
THP exposure increases NETosis in human neutrophils. (**A**) Gating strategy for quantifying neutrophil exDNA^+^ (SYTOX Orange^+^MPO^+^ [P4]) subpopulations of interest focusing on NETosis (Hoechst^var^H3Cit^+^ [P5]) populations that were viable (Live/Dead^–^) or dead (Live/Dead^+^). (**B**–**D**) Frequency for total NETosis (**B**), nonclassical NETosis (**C**), and classical NETosis (**D**). (**E**) Histogram plots depicting H3Cit staining. (**F**) Median fluorescence intensity of H3Cit. Experiments were performed in at least 4 independent experiments and combined, *n* = 7–8 (**B**–**F**). Bar plots show median, all points, and 95% CI (**B**–**D** and **F**). Data were analyzed by 2-way ANOVA with Tukey’s multiple-comparisons test (**B**–**D** and **F**). **P < 0.01; ***P < 0.001; ****P < 0.0001.

To determine if THP effects were specific to cells undergoing NETosis, we assessed the presence of H3Cit via flow cytometry. Neutrophils identified as MPO^+^SO^+^ were further gated based on H3Cit and plasma membrane permeability (Live/Dead stain) as depicted in Figure 8A. We included a caspase inhibitor (YVAD) to test whether blocking other forms of cell death was a potential mechanism of NETosis enhancement. Total NETosis was increased in all PMA-stimulated groups; however, THP further increased proportions of NETosis over PMA and PMA+YVAD groups (Figure 8B). When separated into classical and nonclassical NETosis based on Live/Dead staining, PMA stimulation across all groups significantly increased both forms over mock-stimulated counterparts (Figure 8, C and D). Uniquely, THP further increased nonclassical NETosis over other stimulated groups (Figure 8C). THP-treated, PMA-stimulated cells also displayed higher median fluorescence intensity of H3Cit staining compared with PMA and PMA+YVAD groups (Figure 8, E and F). Inhibition of caspases (YVAD) under these experimental conditions did not alter frequency of NETosis, in line with prior findings (57).

### THP alters NETosis and other forms of cell death as measured by imaging flow cytometry.

**Figure 9:**
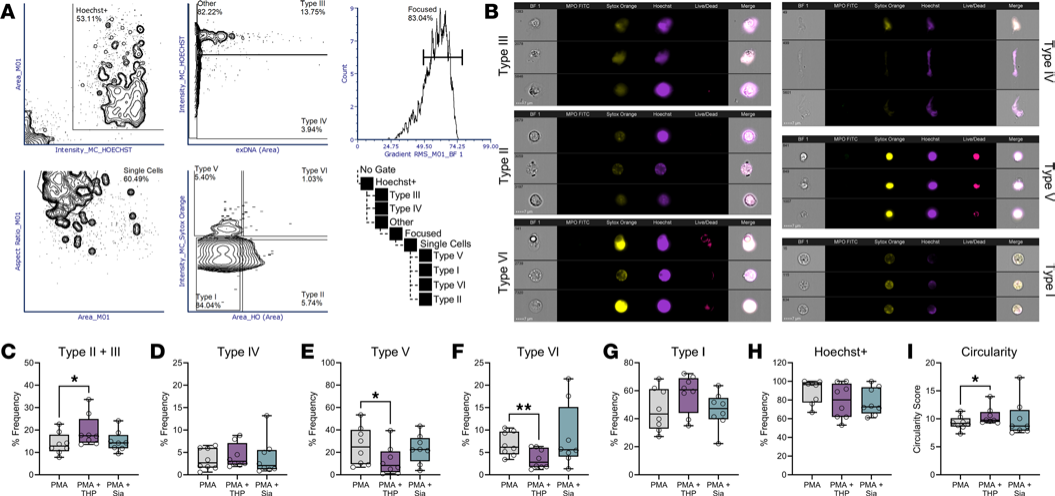
THP exposure alters proportions of NETs, NET precursors, and other cellular morphologies. Neutrophils were stained with anti–MPO-FITC, SYTOX Orange, Hoechst, and Live/Dead stain and visualized for fluorescence and bright-field (BF). (**A** and **B**) Gating strategy (**A**) of neutrophils subpopulations with representative images shown (**B**). (**C**–**G**) Frequency of NETs and NET precursors (Type III and II) (**C**), NET fragments (Type IV) (**D**), dead cells with condensed nuclei (Type V) (**E**), dead cells with decondensed nuclei (Type VI) (**F**), and live cells (Type I) (**G**). (**H**) Frequency of Hoechst^+^ cells. (**I**) Circularity scores across groups. Experiments were performed in 8 experiments and combined, *n* = 8. Box-and-whisker plots show median, all points, and 25–75th percentiles (**C**–**I**). Data were analyzed by 1-way ANOVA with Holm-Šídák’s multiple-comparisons test (**C**–**I**). *P < 0.05. Sia, sialic acid.

Prior to DNA release, cells destined for NETosis undergo cellular remodeling, including cytoskeletal and endoplasmic reticulum disassembly (58), vacuolization, autophagy, superoxide production (57), and lastly, chromatin swelling and nuclear envelope rupture (59). Live cell imaging or imaging flow cytometry techniques have revealed predictable morphologic changes that delineate NETosis from other forms of cell activation and death (22, 47, 58, 60, 61). To assess whether THP altered neutrophil morphology, we adapted an imaging flow cytometry method and algorithm from prior work (60) to identify NETs, NET precursors, and other forms of cell death. Using this method, cells are distinguished into 6 types: healthy (Type I), live cells with decondensed nuclei (NET precursors, Type II), NETs (Type III), DNA NET fragments (Type IV), dead cell condensed nuclei (Type V), and dead cell diffuse nuclei (Type VI). Neutrophils were pretreated with THP or an estimated equivalent amount of sialic acid, stimulated with PMA; stained with α-MPO-FITC, SO, Hoechst 33342, and Live/Dead Near I/R; subjected to imaging flow cytometry; and gated as shown in Figure 9A. Cells were separated from debris based on bright-field (BF) area and Hoechst^+^ staining. Within populations with higher extracellular DNA (SO staining beyond the BF cell margins) area, NETs and DNA NET fragments were distinguished by higher or lower Hoechst intensity, respectively. Remaining cells were further gated to collect focused, single cells and separated based on SO intensity (indicating membrane permeability) and Hoechst area (indicating nuclear area). Dead cells (higher SO intensity) with condensed nuclei or diffuse nuclei were delineated by lower and higher Hoechst area, respectively. Healthy cells and live cells with decondensed nuclei were demarcated by Hoechst area. Representative images of each cell type are shown in Figure 9B with dead cell types (Type V and VI) confirmed by staining Live/Dead^+^. The sum of NET precursors and NETs (Type II and Type III) were higher in THP-treated cells compared with PMA alone, although there were no differences in NET fragments (Type IV; Figure 9, C and D). Additionally, the PMA+THP group exhibited decreased dead cell (Type V and VI) frequencies compared with PMA controls (Figure 9, E and F). Live cell and Hoechst^+^ populations were not different across groups (Figure 9, G and H). We also identified a circularity feature, which gives higher scores to features closely resembling a circle, as significantly higher in PMA+THP compared with PMA alone (Figure 9I). No effects were seen with sialic acid treatment (Figure 9), and no effects of THP were observed in the absence of PMA stimulation, with the exception of a slight but significant decrease in circularity (Supplemental Figure 5, A–G). Overall, these analyses reveal that THP enhances the frequency of NETs and NET precursors in the presence of a NETosis stimulus.

## Discussion

Despite abundant evidence supporting the critical independent roles of THP and neutrophils in protecting against UTI, few studies have investigated direct interactions between these 2 host defenses. Here, we build upon recent findings of THP regulation of neutrophil function and provide characterization of histopathological and immunological consequences of THP deficiency during UTI. From our in vivo experiments, neutrophils emerged as a key cell; THP-deficient mice displayed altered neutrophil proportions and NETosis during UTI. In prior work, we demonstrated that THP sialic acids bind Siglec-9, inhibiting ROS production among other regulatory effects (34). Presently, we show that THP enhances characteristics of NETosis, such as nuclear decondensation and histone citrullination, upon stimulation. We propose that this function, coupled with THP-mediated dampening of excessive neutrophil activation (34), is integral to protecting the urinary tract from infectious and inflammatory insults.

Two independent THP-KO mouse lines have consistently demonstrated that THP deficiency increases susceptibility to UTI (26–29, 62) and aggravates renal pathology (63). Although histologically similar at baseline, THP-KO mice display more severe renal neutrophil infiltration upon kidney injury (35) and increased bladder neutrophil invasion of the uroepithelium during UTI (62). Our findings build upon these prior studies, revealing that THP-KO mice exhibit more severe histopathological alterations in both bladder and renal tissues during UTI. Increased neutrophil recruitment to affected tissues is a primary THP-KO phenotype during injury or exposure to inflammatory stimuli (34, 63). Likewise, we identified neutrophils as the predominant immune cell affected by THP deficiency. In contrast to a prior study (36), we did not observe elevated renal neutrophils in THP-KO mice at baseline; this divergent phenotype may be due to differences between *Neo* cassette placement between the 2 independent THP-KO lines or variations in the methods used to evaluate neutrophils. Neutrophil depletion resolved bacterial burden differences between WT and THP-KO tissues, with neutrophil-depleted WT burdens rising to the level of THP-KO mice. This suggests that neutrophils are dispensable for UTI response in THP-KO mice. We speculate that this may be due to the combined loss of THP-mediated antiadherence (30) and antiinflammatory (34) properties, paired with reduced NETosis demonstrated here, that render the urinary tract highly susceptible to UTI.

Human THP possesses 8 N-glycosylation sites with high-mannose and bi-, tri-, or tetraantennary complex types that are crucial to THP function (31). THP glycosylation facilitates direct interactions with both neutrophils and *E*. *coli* (30, 34). Despite only a 70% identical amino acid sequence, murine THP possesses the same N-glycan sites as humans (64), and here we show that the glycan structures are also similar, including abundant tetraantennary, sialylated, and fucosylated N-glycans. Reduced THP sialic acid levels are reported in patients with UTI, interstitial cystitis, and kidney stones (44, 45, 65); however, we did not observe changes in THP sialic acid levels or total THP during murine UTI. Due to low urine volumes, we pooled multiple mice and collected over 96 hours following infection for glycan analyses. Thus, it is possible that reduction in sialic acid occurs at later periods or that pooling of multiple time points prevented resolving temporary differences. Additionally, we determined that the primary sialic acid on murine THP is Neu5Ac rather than Neu5Gc, a mammalian sialic acid absent in humans (66). Together, these data demonstrate that THP sialic acid levels are sustained during UTI, and they further highlight the utility of mouse models in studying mechanistic functions of THP glycans.

Our visualization of murine urine NETs complements several in vivo studies providing evidence of the importance of NETosis in UTI. A recent study found that PAD4-KO (NETosis-deficient) mice displayed higher bladder and kidney bacterial burdens in UPEC UTI (67). We observed NETs in urine samples but not in bladder and kidney tissues. In line with this, NETs have also been visualized in murine *Proteus mirabilis* UTI in urine and along the luminal surface of the bladder (68). Proteomic urine studies have identified NET-associated proteins in samples from bacterial and fungal UTI, suggesting NETosis may be a conserved urinary defense against a wide range of uropathogens (23, 69). Using label-free methods, urinary neutrophil morphologies consistent with NETosis are visualized in human UTI urine specimens (70). Additionally, NETosis was demonstrated on a UTI bladder-on-a-chip model using urine as the luminal medium; thus, THP would be present in this system (24). These in vitro studies did not distinguish subtypes of NETosis and did not associate NET formation with outcomes. Future work using these platforms could determine the contribution of THP to neutrophil migration, NETosis, and UTI resolution in a dynamic model of the uroepithelium.

We used proteomics to parse signaling pathways affected by THP treatment. Our findings generally aligned with other proteomic-based studies of PMA-induced NETs in human (71) and mouse (72) neutrophils, with some overlap in cellular responses to platelet-activating factor–stimulated NETosis (73). PMA stimulation altered levels of organelle- and cytoskeleton-related proteins which may reflect cytoplasmic changes occurring prior to (58, 59) or during the early, active stages of NETosis (59). We found that THP itself minimally altered protein profiles, with modest increases in tertiary granule and primary lysosome pathways in the presence of PMA. Neutrophil retention of granules may contribute to membrane breakdown during NETosis (74, 75), and autophagy is required for chromatin decondensation (57), suggesting multiple effects of THP exposure. However, there are several factors limiting interpretation. It is possible that measuring changes in protein relative abundances is not the most suitable method with which to evaluate NETosis. Protein translation is dispensable for NETosis, although transcriptional changes are observed as soon as 30 minutes after stimulation (76). Another limitation of our approach is that we evaluated cell pellets that remained after stimulation, thus observing lower levels of NETosis and neutrophil degranulation markers released from activated and/or lysed cells. Additionally, differences in protein kinetics over time were not captured. Even so, by comparing PMA-stimulated cells in the presence or absence of THP, we identified multiple differentially abundant proteins linked to cytoplasmic and chromatin remodeling, and these candidates are the focus of future studies.

Both classical and mitochondrial NETosis are dependent on NOX2 activity (21, 55, 56, 77) and independent of caspase activity (57). NOX-deficient neutrophils fail to induce actin and tubulin polymerization and NET formation (78). Nonclassical NETosis, where the cell membrane initially remains intact, may be NOX independent at early time points but becomes NOX dependent at later time points (79). Nonclassical NETosis is characterized by more extensive histone citrullination, delayed ROS release, dilatation of the nuclear envelope prior to rupture, and presence of exDNA despite having intact plasma membranes (79, 80), and these features were consistently observed in THP-exposed neutrophils in our experiments. THP enhanced NETosis in an ROS-dependent manner and reduced frequency of apoptotic cells and other dead cell morphologies, and these phenotypes could not be explained by sialic acid or caspase activity alone. Although we did not uncover a specific mechanism for THP activity in this study, our observations are similar to prior work demonstrating Siglec-9 crosslinking–mediated nonapoptotic neutrophil death in a ROS-dependent and caspase-independent manner with mitochondrial and ROS involvement (81). It is interesting to speculate that THP, through Siglec-9–mediated alterations to cellular activation, may alter NETosis kinetics under certain stimulations. This would also explain the increased proportion of cells with dilated nuclear envelops and decondensed chromatin in the presence of intact plasma membranes. A limitation of these assays is that they were not performed in the context of human urine or infection; thus, cellular activation may differ. Nonetheless, we still observed similarities in THP-associated increases in nonclassical NETosis in both mouse UTI and human neutrophils in vitro using parallel methodologies.

Imaging flow cytometry has been used to characterize NETosis in various studies. Barbu et al. used nuclear morphology and histone H4 citrullination to assess NETosis (61). Zhao et al. observed populations with diffuse MPO and nuclear staining, indicating decondensed nuclei (suicidal NETosis), and another elongated population with large blebs at 1 pole and nuclear and granular contents at the other, which they termed vital NETosis (82). We did not observe this second morphology, possibly due to differences in time course and stimuli. To differentiate between NETosis and other forms of cell death, Lelliott et al. used a combination of cell permeable and nonpermeable nucleic acid dyes and cell boundaries defined by BF images (60). We adapted this method to our samples and found striking similarities in cell morphologies despite differences in staining (e.g., MPO versus NE, SO versus SYTOX Green, inclusion of Live/Dead viability dye). Observed differences in cell type frequencies were likely due to differences in experimental methods (e.g., we did not use Percoll). Our results further validate this methodology as a robust pipeline for rapidly distinguishing NETs from other forms of cell death. We recommend addition of a Live/Dead stain to confirm cell membrane permeability. It is possible that additional cell types could be distinguished: for instance, while some NETs stained with MPO indicating degranulation, others did not (Supplemental Figure 5H). We found that sialic acid alone did not recapitulate THP effects, suggesting that THP may differentially signal through Siglec-9 compared with free sialic acid or pathogen-mediated engagement of Siglec-9 (83, 84) to alter NETosis.

In summary, this study reveals that THP modulates NETosis in animal models and human neutrophils in vitro. We postulate that this activity provides an additional layer of THP-mediated protection against UTI. In support of our findings, during the revision of this work, another group reported synergistic THP-NET interactions that protected against UTI in a manner dependent on THP glycans (85). Acting as a multifaceted host defense through both blocking pathogen adherence and modulating immune cell function, pharmacologic manipulation of THP may emerge as a promising therapeutic target to improve outcomes and prevent UTI in susceptible populations.

## Methods

### Sex as a biological variable.

This study exclusively examined female mice. It is unknown whether the findings are relevant for male mice. Both male and female human adult donors were included; however, due to small sample sizes, we are underpowered to determine sex-dimorphic effects.

### Bacterial strains and growth conditions.

*E*. *coli* strain UTI89 (86) was grown overnight, shaking, at 37°C in Luria-Bertani (LB) broth. Overnight cultures were resuspended in PBS.

### Murine model of UPEC UTI.

Female WT and THP-KO mice on a 129/sv genetic background (26), 2–5 months of age, were bred at UCSD and BCM. Groups were randomly assigned, and mice were housed 4–5 animals per cage with food and water ad libitum.

Mice were anesthetized with isoflurane and infected transurethrally with 1 × 10^8^ CFU (50 μL) as described (37). One to 10 dpi, urine, bladders, and kidneys were collected. Tissues were homogenized with 1.0 mm–diameter beads (Biospec Products, 11079110z) using a MagNA Lyser (Roche Diagnostics). Serial dilutions of homogenates and urine were plated on LB agar to enumerate CFU. For neutrophil depletion, mice injected i.p. with 10 μg of anti-Ly6G (clone 1A8, BioXCell, BE0075-1) or rat IgG2a isotype (clone 2A3, BioXCell, BE0089) in 100 μL prior to infection. Mice received additional doses on days 2 and 4 after inoculation. Mice were given diclofenac sodium salt (Thermo Fisher Scientific, J62609.06) at an estimated 30 mg/kg/day dose (39) by giving 0.2 mg/mL in drinking water on days 0–6 after inoculation.

### Tissue histopathology.

Tissues were collected at days 1 and 3 after inoculation and fixed in 10% formalin. Tissue sections (4 μm), stained with H&E, were examined by a blinded veterinary pathologist. Histopathology was scored based on number of infiltrating cells, degree of tissue damage, and presence of visible bacteria. Scores ranged from 0 (no lesions) to 4 (severe lesions). Minimal to mild lesions (scores 0–2) consisted of small numbers of infiltrating inflammatory cells and intraluminal bacteria/debris. Severe lesions (scores 3–4) involved fibrinoid necrosis of submucosal blood vessels, submucosal edema, and micropustule formation within the urothelium. BF images were collected using an Echo Revolve microscope at ×400 (bladders) and ×200 (kidneys) magnification.

### Flow cytometry of bladder and kidneys.

Tissues were subjected to flow cytometry as adapted from prior work (12). Cells were blocked with 1:200 mouse BD Fc-block (BD Biosciences) in FACS buffer (1 mM EDTA [Fisher Scientific], 1% FBS [Fisher Scientific], 0.1% sodium azide [VWR International] in PBS) and stained for 30 minutes using the following antibodies (5 μg/mL): anti–CD11b-FITC (clone M1/70, BD Biosciences, 553310), anti–CD11c-PerCP-Cy5.5 (clone N418, eBioscience, 45-0114-82) or anti–CD11c-BV786 (clone N418, BioLegend, 117335), anti–Ly6G-APC (clone 1A8, BioLegend, 127614), anti–MHC-II-APC-Fire750 (clone M5/114.15.2, BioLegend, 107652) or anti–MHC-II-BV650 (clone M5/114.15.2, BD Biosciences, 563415), and anti–CD45-BV510 (clone 30-F11, BioLegend, 103138) or anti-CD45-BV605 (clone 30-F11, BD Pharmingen, 563053). Samples were run on a BD FACSCanto II (BD Biosciences), gated as described (Figure 2A), and analyzed with FlowJo v10.9.0 (FlowJo LLC).

### Urine sediment scoring.

Urine sediment scoring was performed as described (10). Urine was centrifuged (113 *x g*) onto glass slides using a Cytospin 3 (Thermo Fisher Scientific, Shandon). Slides were stained with Wright-Giemsa and visualized by an Olympus BX41 BF microscope at ×200 magnification. PMN cells was calculated from counting 2 independent high-power fields (hpf) and scored by a semiquantitative scoring system: 0, < 1 PMN/hpf; 1, 1–5 PMN/hpf; 2, 6–10 PMN/hpf; 3, 11–20 PMN/hpf; 4, 21–40 PMN/hpf; and 5, > 40 PMN/hpf.

### Flow cytometry of murine urine.

Urine was analyzed by flow cytometry as described (34) with several modifications. Urine volume was recorded and cells were washed and resuspended in 50 μL of FACS buffer. The following antibodies (0.25 μL/sample) and dyes (concentrations indicated) were added: anti–CD11b-FITC (clone M1/70, BD Biosciences, 553310), anti–Ly6G-APC (clone 1A8, BioLegend, 127614), and rabbit anti–Histone H3 (citrulline R2 + R8 + R17) polyclonal antibody (1:100, Abcam, ab5103) conjugated using the PE-Cy7 Conjugation Kit Lightning-Link (Abcam, ab102903), Live/Dead Near IR (1:200, Thermo Fisher Scientific L34975), SO (100 nM, Thermo Fisher Scientific S34861), and Hoechst 33342 (200 nM, Thermo Fisher Scientific 62249). Samples were run on a BD FACSCanto II (BD Biosciences), gated as described (Figure 4A), and analyzed with FlowJo v10.9.0.

### Immunofluorescence of murine urine.

Urine was centrifuged (113 *x* *g*) onto glass slides using a Cytospin 3, fixed and permeabilized, and blocked in PBS+0.01% Tween20 (PBST) with 10% horse serum and 1% BSA. Cells were incubated with primary antibodies: sheep anti-THP polyclonal antibody (1:40, R&D Systems, AF5175) conjugated using the FITC Conjugation Kit Lightning-Link Abcam, ab102884), goat anti-MPO polyclonal antibody (1:200, R&D Systems, AF3667), and rabbit anti–Histone H3 antibody (1:100, Abcam, ab5103) in PBST+1% BSA. Cells were incubated with secondary antibodies anti–goat IgG-AF647 (1:250, Thermo Fisher Scientific, A-21469) and anti–rabbit IgG-Texas Red (1:400, Abcam, ab6800) to visualize MPO and H3Cit respectively. Nuclei were stained with NucBlue Fixed Cell ReadyProbes Reagent (Thermo Fisher Scientific R37606). Fluorescence images were collected using an Echo Revolve microscope at ×600 magnification.

### Tamm-Horsfall glycoprotein purification and quantification.

THP levels of urine (1:5 dilution) was determined by ELISA (Morwell MD Biosciences, M036020) and normalized to creatinine (Crystal Chem, 80350). Samples (54 of 75) falling below the THP limit of detection (2.3 ng/mL) were excluded. All THP-KO samples fell below this limit. THP was purified from human and mouse urine via an adapted protocol (45). Briefly, a diatomaceous earth (DE) slurry was passed through a Büchner funnel to create a DE layer. Urine was filtered through the DE layer, washed with PBS, and dried. THP bound to the layer was solubilized in water. Supernatant containing THP was run through Amicon Ultra 50 kDa filters, and retained protein was measured using BCA assay (Pierce, 23225). THP purity was confirmed by polyacrylamide gel and staining with SimplyBlue SafeStain (Thermo Fisher Scientific. THP was visualized as a single band at ~85 kDa (Supplemental Figure 6).

Human THP was digested using trypsin protease, desalted with C18 stage tips, and measured using the Pierce Quantitative Colorimetric Peptide Assay (Thermo Fisher Scientific, 23275). Tryptic peptides were subjected to liquid chromatography–MS/MS (LC-MS/MS) analysis using a Vanquish Neo UHPLC system coupled to an Orbitrap Eclipse (Thermo Fisher Scientific) mass spectrometer in data-dependent mode with MS1 in Orbitrap and MS/MS in the Ion Trap with HCD fragmentation. The MS data search was carried out in Proteome Discoverer (v2.1, Thermo Fisher Scientific) using Mascot algorithm (v2.4, Matrix Science) against the NCBI human protein database (updated _2021_12_23). Peptides were validated in Percolator with 5% FDR. Gene product inference and quantification were done with label-free iBAQ approach using “gpGrouper” algorithm (87) (Supplemental Table 4).

### THP glycan analyses.

Murine THP was hydrolyzed using 2M acetic acid at 80°C to release sialic acids. Samples were dissolved in water and tagged with DMB reagent, and 2 μg was injected on a Reverse Phase UPLC Florescence Detector set at λex = 373nm, λem = 448nm on a Acquity UPLC BEH C18 column (Waters, 186002350). Solvents included 7% methanol with 0.1% TFA and acetonitrile with 0.1% TFA with a flow rate of 0.4 mL/min. Sialic acids were quantified using Neu5Gc and Neu5Ac standards (Sigma-Aldrich).

N-glycans were released from THP using PNGase-F kit (New England Biolabs, P0709S) and were purified by solid phase extraction using Sep-Pak C18 (1 cc Vac-cartridges, Waters) and poly-graphitized charcoal cartridge (Supelclean Envi-Carb, Supelco). N-glycans were permethylated and analyzed by MALDI-TOF/TOF MS (Bruker, AutoFlex). Dried N-glycans were dissolved in anhydrous DMSO and permethylated using NaOH slurry in anhydrous DMSO and CH3I. Permethylated N-glycans were dissolved in MeOH and mixed in 1:1 (v/v) ratio with Super-DHB (MALDI matrix) before spotting and air-drying. MALDI mass spectral data were acquired in positive and reflectron mode. Data analyses and N-glycan structure annotations were performed using GlycoWork Bench software selecting CFG database.

### Human neutrophil isolation and PicoGreen NETosis assays.

Venous blood was obtained with informed consent from healthy adults. Neutrophils were isolated using PolymorphPrep (Axis-Shield). Fluorescence-based quantification of NETs was performed as described (13). Briefly, neutrophils were seeded in plates at 1 × 10^5^ cells/well, pretreated with 50 μg/mL of THP or mock-treated, and incubated for 30 minutes. Cells were incubated for 3 hours with 25 nM phorbol 12-myristate 13-acetate (PMA; Sigma-Aldrich, P8139) to induce NETosis (13). Micrococcal nuclease was added (500 mU/mL) for 10 minutes, and supernatant was transferred to a new plate. DNA was quantified using a Quant-iT PicoGreen dsDNA Assay Kit (Invitrogen), with fluorescence detection (485 nm excitation, 530 nm emission) by an EnSpire Alpha Multimode Plate Reader (PerkinElmer).

### Human neutrophil proteomics.

Neutrophils were pretreated with THP as above and incubated for an additional 2.5 hours with PMA (25 μM). Cells were pelleted and snap frozen. Protein extraction, digestion, and offline peptide fractionation were performed as described (88). Briefly, cells were lysed and digested using LysC and Trypsin proteases. Peptides were labeled with TMTpro 16 plex isobaric label reagent (Thermo Fisher Scientific) and high-pH offline fractionated to generate 24 peptide pools. Deep-fractionated peptides were separated on an online nanoflow Easy-nLC-1200 system (Thermo Fisher Scientific) and analyzed on Orbitrap Exploris 480 mass spectrometer (Thermo Fisher Scientific). Fractions were loaded on precolumns (2 cm × 100 μm) and separated on in-line 20 cm × 75 μm columns (Reprosil-Pur Basic C18aq) at a flow rate of 200 nL/min over 110 minutes. The MS1, operated in a data-dependent mode with 2-second cycle time, was done in Orbitrap (120,000 resolution, scan range 375–1500 *m/z*, 50 ms injection time) followed by MS2 in Orbitrap at 30,000 resolution (HCD 38%) with TurboTMT algorithm. Dynamic exclusion was set to 20 seconds and the isolation width was set to 0.7 *m/z*. Raw data processing, peptide validation, quantification, and differential analyses were conducted as described (89). Reverse decoys and common contaminants were added to the NCBI refseq human protein database (2020.03.09) using Philosopher (90). Batch correction was performed using ComBat (91) in R package Surrogate Variable Analysis v3.44.0. Group differences were calculated using the moderated 2-tailed *t* test in R package limma (92) using default parameters with exception of robust = True, trend = True, with multiple-hypothesis testing correction via the Benjamini-Hochberg procedure. Normalized data are available in Supplemental Table 3. Gene set enrichment analysis (GSEA) was performed using WebGestalt 2019 (93) with signed log *P* values from limma, and the 8 proteins detected in purified THP were removed prior to analyses. Additional data analysis was performed using R v4.2, Python v3.10, NumPy (94), and Pandas (95).

### Human neutrophil flow cytometry.

Neutrophils were isolated and pretreated with THP as above, Ac-YVAD-cmk at 50 μM (YVAD; Sigma-Aldrich, SML0429), or vehicle-treated, incubated for 30 minutes, and then incubated for 2.5 hours with PMA (25 μM). Cells were stained with the following antibodies (0.25 μL) and dyes (concentrations provided below): anti–MPO-FITC (clone MPO421-8B2, BioLegend, 347201), rabbit anti–Histone H3 antibody (1:100, Abcam, ab5103) conjugated using the PE-Cy7 Conjugation Kit Lightning-Link (Abcam, ab102903), Fc Block (BD Pharmingen, 564219), Live/Dead Aqua (1:200, Thermo Fisher Scientific, L34957), SO (100 nM, Thermo Fisher Scientific, S34861), and Hoechst 33342 (200 nM, Thermo Fisher Scientific, 62249). Samples were run on a BD LSRII (BD Biosciences), gated (10,000 cells/sample for Figure 7; 20,000 cells/sample for Figure 8) as described in Figure 7A and Figure 8A, and analyzed with FlowJo v10.9.0.

### Imaging flow cytometry.

Neutrophils were isolated as above and pretreated with THP (80 μg/mL), pretreated with sialic acid (500 ng/mL, Sigma-Aldrich, A0812), or mock-treated, incubated for 30 minutes, and then incubated for 2.5 hours with PMA (25 μM). The following antibodies (0.5 μg/mL) and dyes (concentrations indicated) were added: anti–MPO-FITC (clone MPO421-8B2, BioLegend, 347201), Fc Block, Live/Dead Near IR (1:200, Thermo Fisher Scientific L34975), SO (100 nM, Thermo Fisher Scientific S34861), and Hoechst 33342 (200 nM, Thermo Fisher Scientific 62249). Cell morphology was assessed as described previously (60). An Amnis ImageStream X Mark II imaging flow cytometer was used for data acquisition with a 60× objective, low flow rate, high sensitivity, and 405, 488, 561, and 635 nm lasers set to 120, 150, 100, and 150 mW, respectively. Data were analyzed using the IDEAS v6.3. Clipped images were retained, and single-stained controls were used for compensation. Masking was performed using the default “object (tight)” and “morphology” algorithms (60). Statistics reports were generated using IDEAS and analyzed in FCS Express 7.

### Statistics.

Data were collected from at least 2 independent experiments unless indicated otherwise. Mean values from independent experiment replicates, or biological replicates, are represented by medians with interquartile ranges, or as box-and-whisker plots, with experimental samples size (*n*) indicated in figure legends. For histopathology and urine sediment scores, mice were grouped into low (scores 0–2) or high (scores >2) categories and compared by Fisher’s exact test. Urine and tissue burdens and THP urine levels were analyzed using Mann-Whitney *U* test followed by multiple comparisons correction using the Holm-Šídák method where applicable. Immune cell populations, neutrophil depletion experiments, and flow cytometry of human and mouse neutrophil populations were analyzed using 2-way ANOVA with Šídák’s or Tukey’s multiple-comparisons tests, or uncorrected Fisher’s Least Significant Difference (LSD) test as indicated in figure legends. Imaging flow cytometry populations and murine sialic acid levels were compared using 1-way ANOVA with Holm-Šídák’s multiple-comparisons test. Proteomics data were analyzed by moderated 2-tailed *t* test followed by multiple-hypothesis testing correction using the Benjamini-Hochberg procedure with FDR < 0.05. Statistical analyses were performed using GraphPad Prism, v10.0.2. *P* < 0.05 was considered statistically significant.

### Study approval.

Human blood and urine were obtained from healthy adult volunteers with written, informed consent under approval from UCSD IRB (no. 131002) and BCM IRB (no. H-47537). Animal protocols and procedures were approved by UCSD and BCM IACUCs under protocol nos. S00227M and AN-8233, respectively.

### Data availability.

MS proteomics data (identifier no. PXD045468) are deposited to the ProteomeXchange Consortium (https://www.ebi.ac.uk/pride/archive/projects/PXD045468). ImageStream data are deposited in Figshare under project “ISX data files for THP NETosis Manuscript” (https://doi.org/10.6084/m9.figshare.25013786, https://doi.org/10.6084/m9.figshare.25013774, https://doi.org/10.6084/m9.figshare.25013744, https://doi.org/10.6084/m9.figshare.26496736, https://doi.org/10.6084/m9.figshare.26496304, https://doi.org/10.6084/m9.figshare.26496820, https://doi.org/10.6084/m9.figshare.26496889, and https://doi.org/10.6084/m9.figshare.25013651). Additional source data are provided in the accompanying Supporting Data Values file.

## Author contributions

Research studies were designed by VN and KAP. VME, HB, CC, CS, MEM, JJZ, and KAP conducted experiments and acquired data. Data were analyzed by CC, ABS, IC, and KAP. VME and KAP drafted the manuscript, and all authors contributed to manuscript edits. Order of co–first authors was determined by chronological order of working on the project.

## Supplementary Material

Supplemental data

Unedited blot and gel images

Supplemental table 3

Supplemental table 4

Supporting data values

## Figures and Tables

**Table 1:**
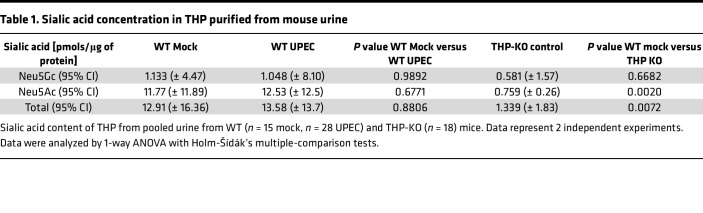
Sialic acid concentration in THP purified from mouse urine

## Version 1 11/26/2024

In-Press Preview

## Version 2 01/09/2025

Electronic publication

## References

[1] Yang X (2022). Disease burden and long-term trends of urinary tract infections: a worldwide report. Front Public Health.

[2] Foxman B (2014). Urinary tract infection syndromes: occurrence, recurrence, bacteriology, risk factors, and disease burden. Infect Dis Clin North Am.

[3] Ambite I (2016). Susceptibility to urinary tract infection: benefits and hazards of the antibacterial host response. Microbiol Spectr.

[4] Chu CM, Lowder JL (2018). Diagnosis and treatment of urinary tract infections across age groups. Am J Obstet Gynecol.

[5] Bergsten G (2004). PapG-dependent adherence breaks mucosal inertia and triggers the innate host response. J Infect Dis.

[6] Sundac L (2016). Protein-based profiling of the immune response to uropathogenic Escherichia coli in adult patients immediately following hospital admission for acute cystitis. Pathog Dis.

[7] Isaacson B (2017). Stromal cell-derived Factor 1 mediates immune cell attraction upon urinary tract infection. Cell Rep.

[8] Ingersoll MA (2008). G-CSF induction early in uropathogenic Escherichia coli infection of the urinary tract modulates host immunity. Cell Microbiol.

[9] Svensson M (2005). Natural history of renal scarring in susceptible mIL-8Rh^–/–^ mice. Kidney Int.

[10] Hannan TJ (2014). Inhibition of Cyclooxygenase-2 Prevents chronic and recurrent cystitis. EBioMedicine.

[11] Chromek M (2006). The antimicrobial peptide cathelicidin protects the urinary tract against invasive bacterial infection. Nat Med.

[12] Patras KA (2020). Host cathelicidin exacerbates group B *Streptococcus* urinary tract infection. mSphere.

[13] Patras KA (2019). Augmentation of urinary lactoferrin enhances host innate immune clearance of uropathogenic Escherichia coli. J Innate Immun.

[14] Schiwon M (2014). Crosstalk between sentinel and helper macrophages permits neutrophil migration into infected uroepithelium. Cell.

[15] Gupta A (1996). Reactive oxygen species-mediated tissue injury in experimental ascending pyelonephritis. Kidney Int.

[16] Condron C (2003). Neutrophil bactericidal function is defective in patients with recurrent urinary tract infections. Urol Res.

[17] Brinkmann V (2004). Neutrophil extracellular traps kill bacteria. Science.

[18] Metzler KD (2011). Myeloperoxidase is required for neutrophil extracellular trap formation: implications for innate immunity. Blood.

[19] Hoppenbrouwers T (2017). In vitro induction of NETosis: Comprehensive live imaging comparison and systematic review. PLoS One.

[20] Tan C (2021). The vitals of NETs. J Leukoc Biol.

[21] Yousefi S (2009). Viable neutrophils release mitochondrial DNA to form neutrophil extracellular traps. Cell Death Differ.

[22] Yipp BG (2012). Infection-induced NETosis is a dynamic process involving neutrophil multitasking in vivo. Nat Med.

[23] Yu Y (2019). Detection of neutrophil extracellular traps in urine. Methods Mol Biol.

[24] Sharma K (2021). Dynamic persistence of UPEC intracellular bacterial communities in a human bladder-chip model of urinary tract infection. Elife.

[25] Liu Y (2018). Tamm-Horsfall protein/uromodulin deficiency elicits tubular compensatory responses leading to hypertension and hyperuricemia. Am J Physiol Renal Physiol.

[26] Bates JM (2004). Tamm-Horsfall protein knockout mice are more prone to urinary tract infection: rapid communication. Kidney Int.

[27] Mo L (2004). Ablation of the Tamm-Horsfall protein gene increases susceptibility of mice to bladder colonization by type 1-fimbriated Escherichia coli. Am J Physiol Renal Physiol.

[28] Raffi HS (2009). Tamm-horsfall protein protects against urinary tract infection by proteus mirabilis. J Urol.

[29] Coady A (2018). Tamm-Horsfall protein protects the urinary tract against *Candida albicans*. Infect Immun.

[30] Pak J (2001). Tamm-Horsfall protein binds to type 1 fimbriated Escherichia coli and prevents E. coli from binding to uroplakin Ia and Ib receptors. J Biol Chem.

[31] Weiss GL (2020). Architecture and function of human uromodulin filaments in urinary tract infections. Science.

[32] Yu CL (1992). Tamm-Horsfall glycoprotein (THG) purified from normal human pregnancy urine increases phagocytosis, complement receptor expressions and arachidonic acid metabolism of polymorphonuclear neutrophils. Immunopharmacology.

[33] Su SJ, Yeh TM (1999). The dynamic responses of pro-inflammatory and anti-inflammatory cytokines of human mononuclear cells induced by uromodulin. Life Sci.

[34] Patras KA (2017). Tamm-Horsfall glycoprotein engages human Siglec-9 to modulate neutrophil activation in the urinary tract. Immunol Cell Biol.

[35] El-Achkar TM (2008). Tamm-Horsfall protein protects the kidney from ischemic injury by decreasing inflammation and altering TLR4 expression. Am J Physiol Renal Physiol.

[36] Micanovic R (2015). Tamm-Horsfall protein regulates granulopoiesis and systemic neutrophil homeostasis. J Am Soc Nephrol.

[37] Zulk JJ (2022). Phage resistance accompanies reduced fitness of uropathogenic Escherichia coli in the urinary environment. mSphere.

[38] Dou W (2005). Defective expression of Tamm-Horsfall protein/uromodulin in COX-2-deficient mice increases their susceptibility to urinary tract infections. Am J Physiol Renal Physiol.

[39] Mayorek N (2010). Diclofenac inhibits tumor growth in a murine model of pancreatic cancer by modulation of VEGF levels and arginase activity. PLoS One.

[40] Ghirotto S (2016). The uromodulin gene locus shows evidence of pathogen adaptation through human evolution. J Am Soc Nephrol.

[41] Garimella PS (2017). Urinary uromodulin and risk of urinary tract infections: the cardiovascular health study. Am J Kidney Dis.

[42] https://atcmeetingabstracts.com/abstract/reduced-urinary-uromodulin-umod-levels-are-associated-with-urinary-tract-infections-uti-after-renal-transplantion.

[43] Reinhart HH (1992). Quantitation of urinary Tamm-Horsfall protein in children with urinary tract infection. Eur Urol.

[44] Parsons CL (2011). A multi-site study confirms abnormal glycosylation in the Tamm-Horsfall protein of patients with interstitial cystitis. J Urol.

[45] Argade S (2015). An evaluation of Tamm-Horsfall protein glycans in kidney stone formers using novel techniques. Urolithiasis.

[46] Li H (2021). Uromodulin isolation and its *N*-Glycosylation analysis by NanoLC-MS/MS. J Proteome Res.

[47] Singhal A (2022). An imaging and computational algorithm for efficient identification and quantification of neutrophil extracellular Traps. Cells.

[48] Zharkova O (2019). A flow cytometry-based assay for high-throughput detection and quantification of neutrophil extracellular traps in mixed cell populations. Cytometry A.

[49] Masuda S (2017). Measurement of NET formation in vitro and in vivo by flow cytometry. Cytometry A.

[50] Perfetto SP (2010). Amine-reactive dyes for dead cell discrimination in fixed samples. Curr Protoc Cytom.

[51] Yousefi S (2020). In vivo evidence for extracellular DNA trap formation. Cell Death Dis.

[52] Vorobjeva NV, Chernyak BV (2020). NETosis: molecular mechanisms, role in physiology and pathology. Biochemistry (Mosc).

[53] Wang Y (2009). Histone hypercitrullination mediates chromatin decondensation and neutrophil extracellular trap formation. J Cell Biol.

[54] Swensen AC (2021). A comprehensive urine proteome database generated from patients with various renal conditions and prostate cancer. Front Med (Lausanne).

[55] Parker H (2012). Requirements for NADPH oxidase and myeloperoxidase in neutrophil extracellular trap formation differ depending on the stimulus. J Leukoc Biol.

[56] Fuchs TA (2007). Novel cell death program leads to neutrophil extracellular traps. J Cell Biol.

[57] Remijsen Q (2011). Neutrophil extracellular trap cell death requires both autophagy and superoxide generation. Cell Res.

[58] Thiam HR (2020). NETosis proceeds by cytoskeleton and endomembrane disassembly and PAD4-mediated chromatin decondensation and nuclear envelope rupture. Proc Natl Acad Sci U S A.

[59] Neubert E (2018). Chromatin swelling drives neutrophil extracellular trap release. Nat Commun.

[60] Lelliott PM (2019). Rapid Quantification of NETs in vitro and in whole blood samples by imaging flow cytometry. Cytometry A.

[61] Barbu EA (2020). Detection and quantification of histone H4 citrullination in early NETosis with image flow cytometry version 4. Front Immunol.

[62] Raffi HS (2005). Tamm-Horsfall protein acts as a general host-defense factor against bacterial cystitis. Am J Nephrol.

[63] El-Achkar TM (2011). Tamm-Horsfall protein-deficient thick ascending limbs promote injury to neighboring S3 segments in an MIP-2-dependent mechanism. Am J Physiol Renal Physiol.

[64] Prasadan K (1995). Nucleotide sequence and peptide motifs of mouse uromodulin (Tamm-Horsfall protein) — the most abundant protein in mammalian urine. Biochim Biophys Acta.

[65] Olczak T (1999). Composition of the sugar moiety of Tamm-Horsfall protein in patients with urinary diseases. Int J Clin Lab Res.

[66] Hedlund M (2007). N-glycolylneuraminic acid deficiency in mice: implications for human biology and evolution. Mol Cell Biol.

[67] Krivosikova K (2023). Neutrophil extracellular traps in urinary tract infection. Front Pediatr.

[68] Schaffer JN (2016). Proteus mirabilis fimbriae- and urease-dependent clusters assemble in an extracellular niche to initiate bladder stone formation. Proc Natl Acad Sci U S A.

[69] Yu Y (2017). Characterization of early-phase neutrophil extracellular traps in urinary tract infections. PLoS Pathog.

[70] Petchakup C (2023). Rapid screening of urinary tract infection using microfluidic inertial-impedance cytometry. ACS Sens.

[71] Petretto A (2019). Neutrophil extracellular traps (NET) induced by different stimuli: a comparative proteomic analysis. PLoS One.

[72] Wang X (2018). A label-free quantitative proteomic analysis of mouse neutrophil extracellular trap formation induced by *Streptococcus suis* or phorbol myristate acetate (PMA). Front Immunol.

[73] Aquino EN (2016). Proteomic analysis of neutrophil priming by PAF. Protein Pept Lett.

[74] Aarts CEM (2021). Neutrophil specific granule and NETosis defects in gray platelet syndrome. Blood Adv.

[75] Tao M (2023). Identification and validation of immune-associated NETosis subtypes and biomarkers in anti-neutrophil cytoplasmic antibody associated glomerulonephritis. Front Immunol.

[76] Khan MA, Palaniyar N (2017). Transcriptional firing helps to drive NETosis. Sci Rep.

[77] Bjornsdottir H (2015). Neutrophil NET formation is regulated from the inside by myeloperoxidase-processed reactive oxygen species. Free Radic Biol Med.

[78] Stojkov D (2017). ROS and glutathionylation balance cytoskeletal dynamics in neutrophil extracellular trap formation. J Cell Biol.

[79] Pilsczek FH (2010). A novel mechanism of rapid nuclear neutrophil extracellular trap formation in response to Staphylococcus aureus. J Immunol.

[80] Douda DN (2015). SK3 channel and mitochondrial ROS mediate NADPH oxidase-independent NETosis induced by calcium influx. Proc Natl Acad Sci U S A.

[81] von Gunten S (2005). Siglec-9 transduces apoptotic and nonapoptotic death signals into neutrophils depending on the proinflammatory cytokine environment. Blood.

[82] Zhao W (2015). A novel image-based quantitative method for the characterization of NETosis. J Immunol Methods.

[83] Secundino I (2016). Host and pathogen hyaluronan signal through human siglec-9 to suppress neutrophil activation. J Mol Med (Berl).

[84] Khatua B (2012). Sialoglycoproteins adsorbed by Pseudomonas aeruginosa facilitate their survival by impeding neutrophil extracellular trap through siglec-9. J Leukoc Biol.

[85] Stewart AP (2024). Neutrophil extracellular traps protect the kidney from ascending infection and are required for a positive leukocyte dipstick test. Sci Transl Med.

[86] Mulvey MA (2001). Establishment of a persistent Escherichia coli reservoir during the acute phase of a bladder infection. Infect Immun.

[87] Saltzman AB (2018). gpGrouper: a peptide grouping algorithm for gene-centric inference and quantitation of bottom-up proteomics data. Mol Cell Proteomics.

[88] Mertins P (2014). Ischemia in tumors induces early and sustained phosphorylation changes in stress kinase pathways but does not affect global protein levels. Mol Cell Proteomics.

[89] Nozawa K (2023). Testis-specific serine kinase 3 is required for sperm morphogenesis and male fertility. Andrology.

[90] da Veiga Leprevost F (2020). Philosopher: a versatile toolkit for shotgun proteomics data analysis. Nat Methods.

[91] Johnson WE (2007). Adjusting batch effects in microarray expression data using empirical Bayes methods. Biostatistics.

[92] Ritchie ME (2015). Limma powers differential expression analyses for RNA-sequencing and microarray studies. Nucleic Acids Res.

[93] Liao Y (2019). WebGestalt 2019: gene set analysis toolkit with revamped UIs and APIs. Nucleic Acids Res.

[94] Harris CR (2020). Array programming with NumPy. Nature.

[95] https://github.com/pandas-dev/pandas/tree/v2.0.0rc0.

