# Supplementary material for: Tamm-Horsfall protein augments neutrophil NETosis during urinary tract infection
Source: JCI Insight. 2025 Jan 9;10(1):e180024. doi: 10.1172/jci.insight.180024 (PMC11721310; doi:10.1172/jci.insight.180024)
Supplement: Supplemental data [file jciinsight-10-180024-s199.pdf]

SUPPLEMENTAL MATERIAL

Tamm-Horsfall protein augments neutrophil NETosis during urinary tract infection

Vicki Mercado-Evans, Holly Branthoover, Claude Chew, Camille Serchejian,
Alexander B. Saltzman, Marlyd E. Mejia, Jacob J. Zulk, Ingrid Cornax,
Victor Nizet, Kathryn A. Patras

Contents:

Supplementary Figures 1-6

Supplementary Tables 1-2

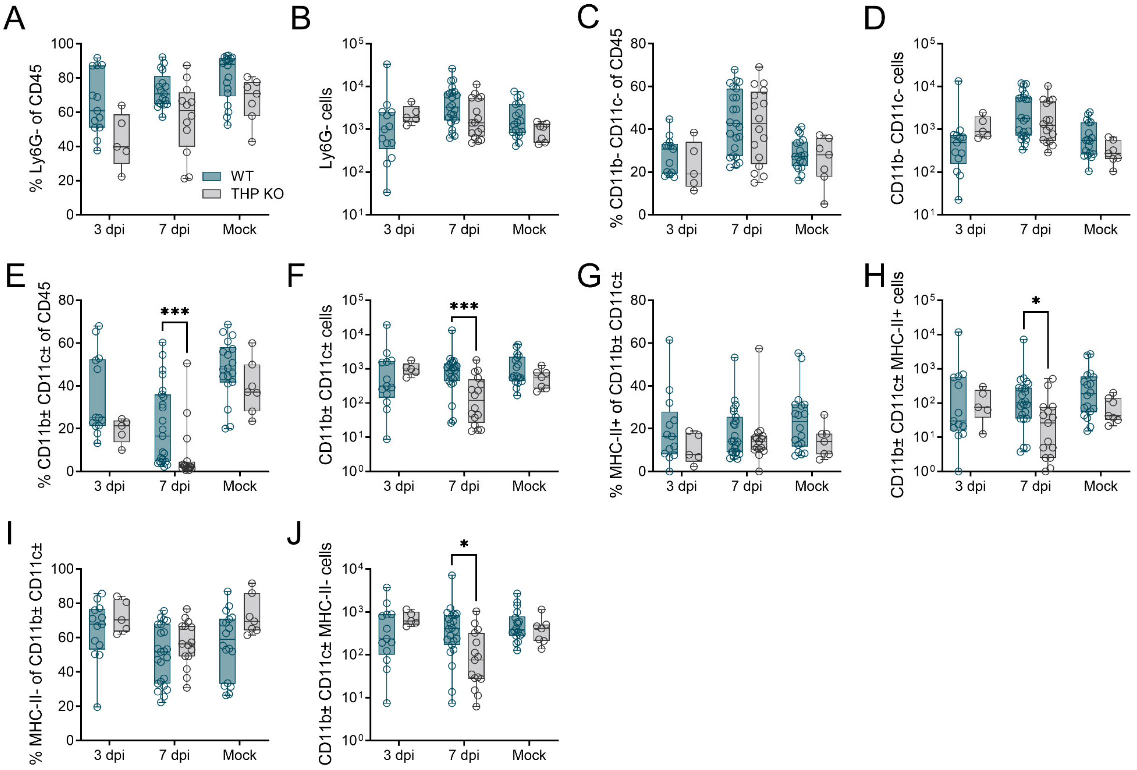

Supplemental Figure 1. Impact of THP on other bladder immune populations during UTI. Wild type (WT) and THP knockout (THP KO) mice were transurethrally infected with 10^8 CFU of UPEC strain UTI89 or mock-infected as a control. Frequency (**A**) and total counts (**B**) of Ly6G- cells, frequency (**C**) and counts (**D**) of CD11b-CD11c- (lymphocytes) cells, and frequency (**E**) and counts (**F**) $CD11b^{var}CD11c^{var}$ (myeloid) cells. Frequency (**G**) and total counts (**H**) of MHC-II+ (antigen presenting cells) and frequency (**I**) and counts (**J**) of MHC-II- (other myeloid) cells. Frequency is expressed as a percentage of total bladder CD45+ cells (A, C, E) or as a percentage of total myeloid ($CD11b^{var}CD11c^{var}$) cells (G, I). Experiments were performed at least two times with data combined, *n* = 5-23/group. Box and whisker plots extend from 25th to 75th percentiles, horizontal bars indicate median, and all points are shown. Data were analyzed by Mann-Whitney *U* test with Holm-Šídák's multiple comparisons correction. **P*<0.05, ****P*<0.001. Supplemental data to **Figure 2**.

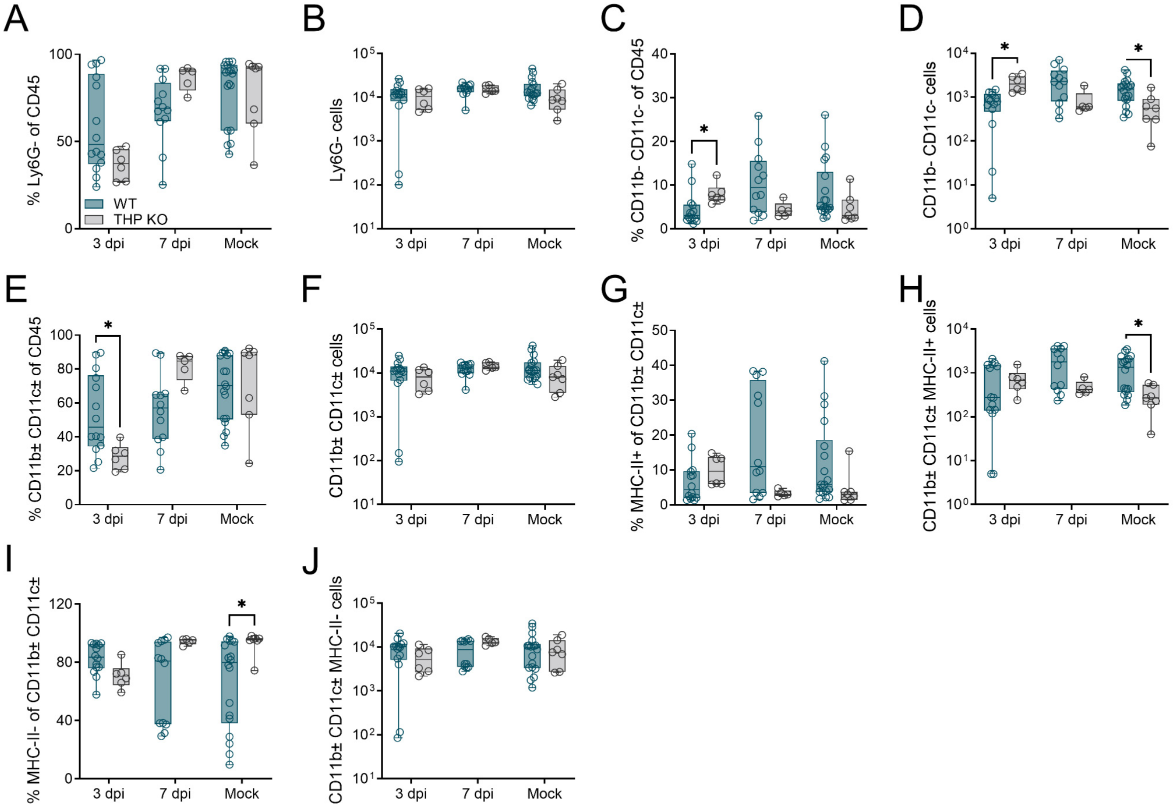

Supplemental Figure 2. Impact of THP on other kidney immune populations during UTI. Wild type (WT) and THP knockout (THP KO) mice were transurethrally infected with 10^8 CFU of UPEC strain UTI89 or mock-infected as a control. Frequency (**A**) and total counts (**B**) of Ly6G- cells, frequency (**C**) and counts (**D**) of CD11b-CD11c- (lymphocytes) cells, and frequency (**E**) and counts (**F**) $CD11b^{var}CD11c^{var}$ (myeloid) cells. Frequency (**G**) and total counts (**H**) of MHC-II+ (antigen presenting cells) and frequency (**I**) and counts (**J**) of MHC-II- (other myeloid) cells. Frequency is expressed as a percentage of total kidney CD45+ cells (A, C, E) or as a percentage total myeloid ($CD11b^{var}CD11c^{var}$) cells (G, I). Experiments were performed at least two times with data combined, n = 5-18/group. Box and whisker plots extend from 25th to 75th percentiles, horizontal bars indicate median, and all points are shown. Data were analyzed by Mann-Whitney *U* test with Holm-Šídák's multiple comparisons correction. *$P<0.05$. Supplemental data to **Figure 2**.

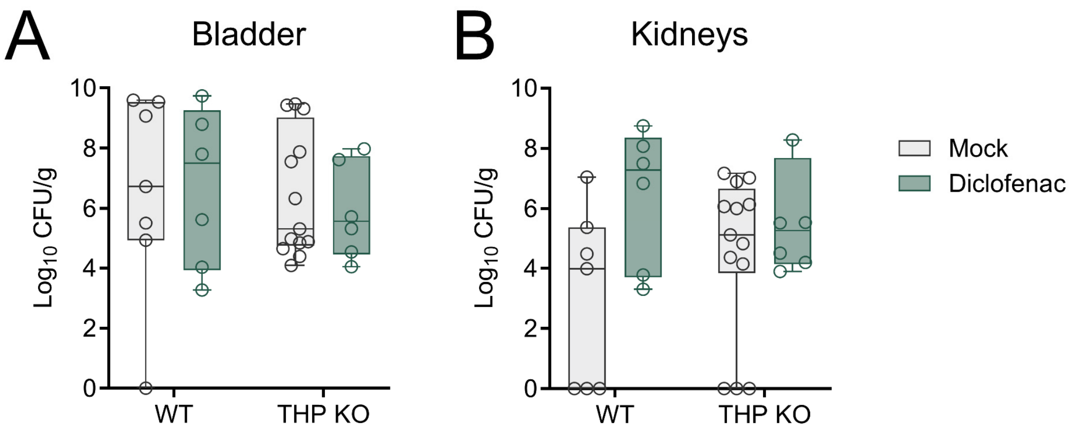

Supplemental Figure 3. Cox-2 inhibitor Diclonfenac does not alter bacterial burdens in WT or THP KO mice. Wild type (WT) and THP knockout (THP KO) mice were transurethrally infected with 10^8 CFU of UPEC strain UTI89. To inhibit COX-2, mice were administered diclofenac in the drinking water beginning on day 0 and maintained through day 6 post-infection. Bladder (**A**) and kidney (**B**) UPEC burdens at 7 days post-infection. Experiments were performed at least two times with data combined, n = 6-13/group. Box and whisker plots extend from 25th to 75th percentiles, horizontal bars indicate median, and all points are shown. Data were analyzed by two-way ANOVA with Sidak’s multiple comparisons test and all comparisons were determine not significant (P>0.05). Supplemental data to **Figure 2**.

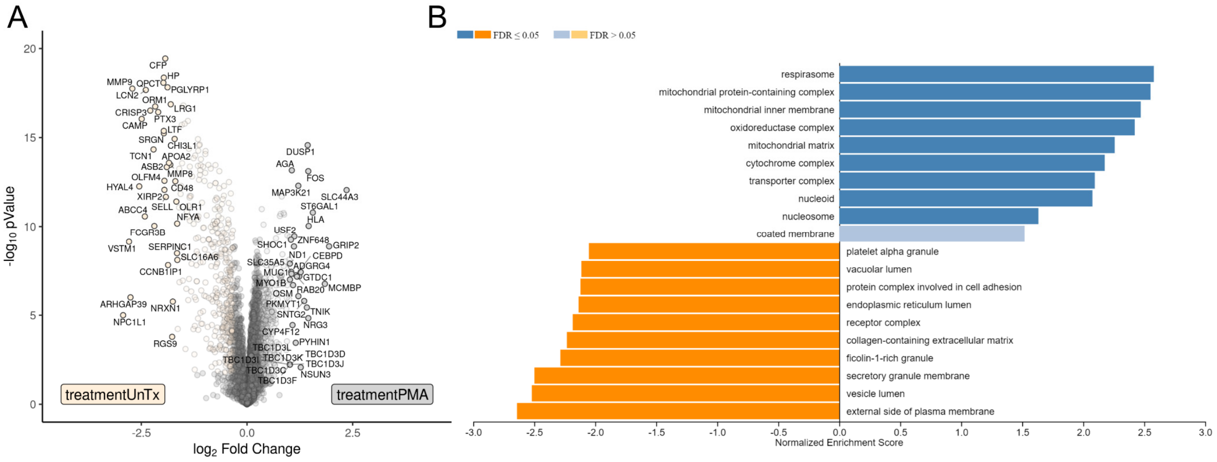

Supplemental Figure 4. PMA stimulation alters the neutrophil proteome. Peripheral human neutrophils were isolated, pretreated with 50 μg/mL THP, and stimulated with phorbol 12-myristate 13-acetate (PMA) for 2.5 hours. Volcano plot (**A**) and gene set enrichment analysis (**B**) of differentially identified proteins in untreated vs. PMA stimulated samples. Experiments were performed as part of one independent experiment, *n* = 4 donors. Differential proteins were identified via Log_2 fold change >1.25 and moderated t-test followed by multiple-hypothesis testing correction using the Benjamini–Hochberg procedure with a false discovery rate adjusted P <0.05. GSEA was performed with a gene set minimum of 10, a gene set maximum of 500, 2,000 permutations using the gene ontology cellular component gene sets. Supplemental data to proteomic analyses in **Figure 6**.

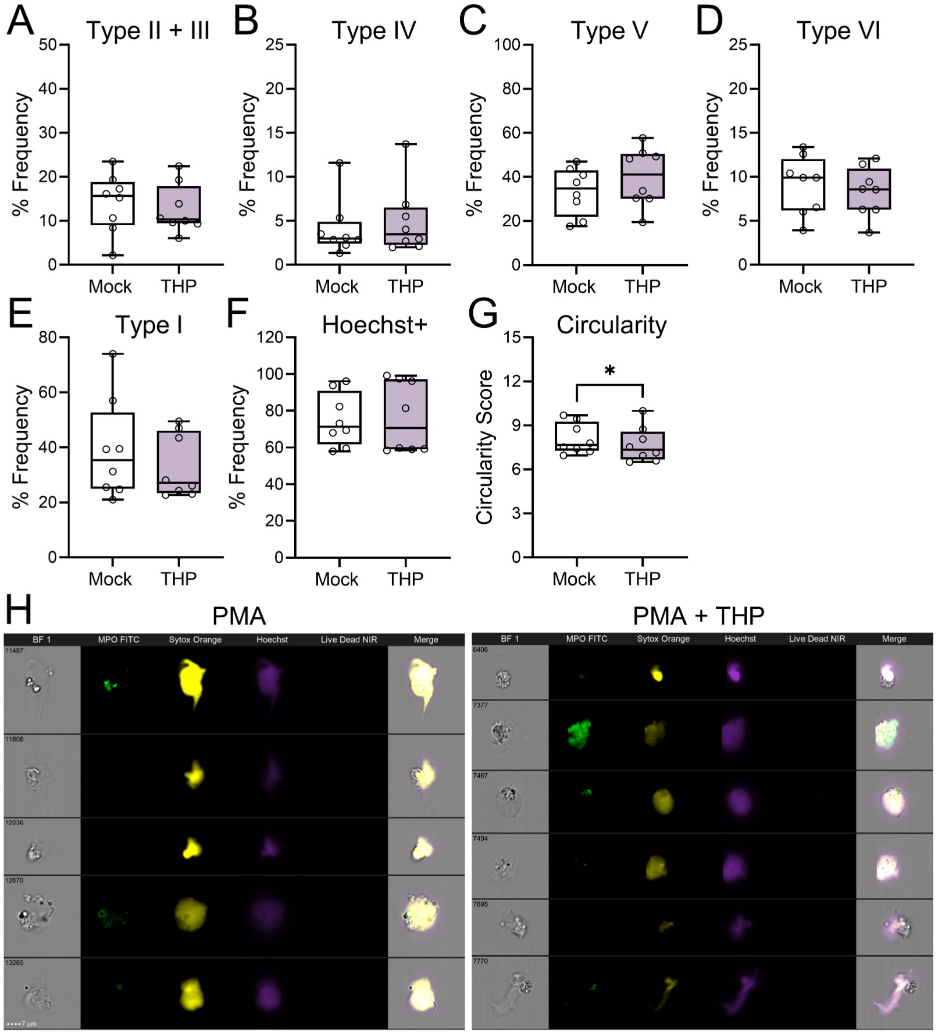

Supplemental Figure 5. Additional effects of THP on cellular morphologies as determined by imaging flow cytometry. Peripheral human neutrophils were isolated, pretreated with THP and mock-stimulated (A-G) or stimulated with phorbol 12-myristate 13-acetate (PMA) for 2.5 hours (H). Cells were stained with anti-MPO FITC, Sytox Orange (non-membrane permeable nucleic acid dye), Hoechst (membrane-permeable nucleic acid dye), and Live/Dead stain (non-membrane permeable amine-reactive dye) and visualized for fluorescence and brightfield (BF) images on an imaging flow cytometer. Frequency of NETs and NET precursors (Type III and II) (**A**), NETs fragments (Type IV) (**B**), dead cells with condensed nuclei (Type V) (**C**), dead cells with decondensed nuclei (Type VI) (**D**), and live cells (Type I) (**E**). (**F**) Frequency of Hoechst+ cells. (**G**) Circularity scores (degree of the Hoechst mask deviation from a circle, with lower values having more deviation) across groups. (**H**) Additional representative images of Type III NETs from PMA and PMA + THP treatment groups showing variable levels of MPO staining across groups. Experiments were performed in eight independent experiments with data combined, n = 8 donors. Box and whisker plots extend from 25th to 75th percentiles, horizontal bars indicate median, and all points are shown (A-G). Data were analyzed by Mann-Whitney U test (A-G). *P<0.05. Supplemental data to **Figure 9**.

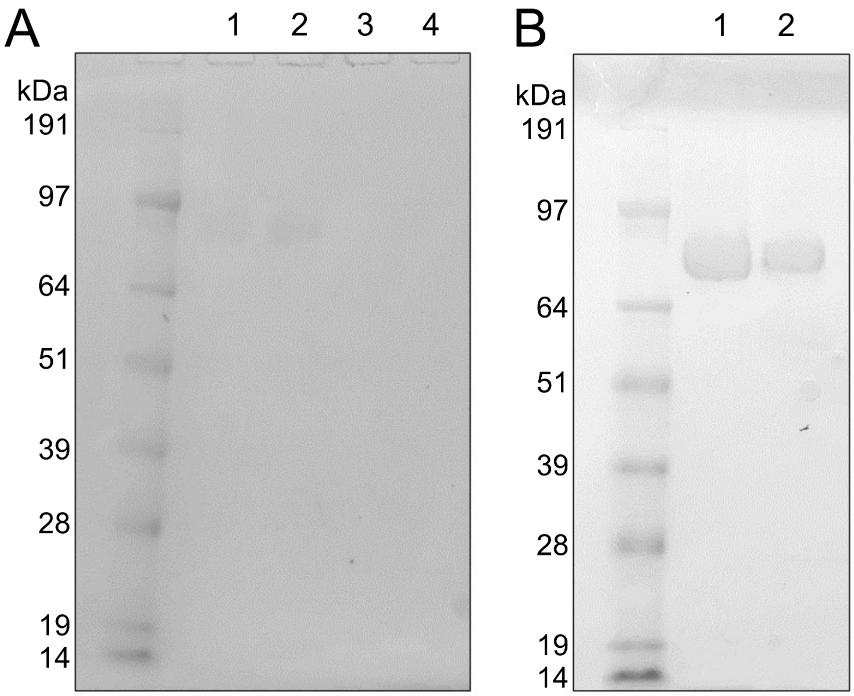

Supplemental Figure 6. Representative protein gels of THP isolated from mouse and human urine. (**A**) Polyacrylamide protein gel of THP isolated from pooled mouse urine as described in Methods. Lane 1: WT mock-infected, Lane 2: WT UPEC-infected, Lane 3: THP KO mock-infected, Lane 4: THP KO UPEC-infected. THP was visualized as a single band at ~ 85 kDa in lanes 1 and 2. (**B**) Polyacrylamide protein gel of THP isolated from pooled human urine as described in Methods. Lane 1: pooled batch #1, Lane 2: pooled batch #2. THP was visualized as a single band at ~ 85 kDa in lanes 1 and 2.

Supplemental Table 1. Comparative N-glycan MALDI-tof analyses of THP during UPEC infection. N-glycan MALDI-tof peaks of THP purified from urine of WT mice that were either mock-infected or UPEC-infected. Data represent one MALDI-tof analysis of pooled purified THP harvested from two independent experiments. Supplemental data to **Figure 3**.

m/z peak	WT Mock	WT UPEC	% change
2244.459	1.93	1.31	-47.1
2431.552	4.24	3.59	-18.1
2605.648	8.40	9.00	6.6
2792.735	2.47	2.75	10.2
2966.841	14.28	17.17	16.9
3241.968	4.00	4.46	10.1
3416.062	8.41	13.05	35.6
3777.244	17.42	17.32	-0.5
4226.453	14.22	16.24	12.5
4587.622	24.64	15.11	-63.1

Supplemental table 2. Differentially abundant proteins between THP-treated and mock-treated human neutrophils in unstimulated and PMA-stimulated conditions. Supplemental data to **Figure 6**.

Gene ID	Gene Symbol	Gene Description	Log$_2$ Fold-Change Unstimulated[a] (*p*-value)[c]	Log$_2$ Fold-Change Stimulated[b] (*p*-value)
7369	UMOD[d]	uromodulin	2.894 **(2.14E-12)**	2.497 **(3.77E-11)**
347	APOD[d]	apolipoprotein D	2.112 **(2.14E-12)**	1.200 **(6.47E-09)**
55902	ACSS2	acyl-CoA synthetase short-chain family member 2	0.684 **(7.73E-08)**	0.466 **(1.27E-05)**
23506	BICRAL	GLTSCR1-like	0.669 **(0.039)**	0.032 (0.962)
3959	LGALS3BP[d]	lectin, galactoside-binding, soluble, 3 binding protein	0.624 **(2.70E-09)**	0.533 **(2.62E-08)**
3827	KNG1[d]	kininogen 1	0.612 **(7.79E-07)**	0.485 **(1.58E-05)**
2731	GLDC	glycine dehydrogenase (decarboxylating)	0.539 **(0.017)**	0.445 (0.052)
259	AMBP[d]	alpha-1-microglobulin/bikunin precursor	0.488 **(3.02E-05)**	0.647 **(1.47E-06)**
220963	SLC16A9	solute carrier family 16, member 9	0.485 **(4.93E-04)**	0.364 **(0.005)**
3561	IL2RG	interleukin 2 receptor, gamma	0.456 **(3.02E-05)**	0.325 **(0.001)**
10621	POLR3F	polymerase (RNA) III (DNA directed) polypeptide F, 39 kDa	0.422 **(0.030)**	0.184 (0.445)
952	CD38	CD38 molecule	0.382 **(0.017)**	0.142 (0.487)
55647	RAB20	RAB20, member RAS oncogene family	0.554 **(0.046)**	0.088 (0.848)
440275	EIF2AK4	eukaryotic translation initiation factor 2 alpha kinase 4	0.352 **(1.66E-06)**	0.201 (0.001)
107987285	LOC107987285	uncharacterized protein	0.344 **(0.042)**	0.030 (0.928)
8225	GTPBP6	GTP binding protein 6 (putative)	0.221 (0.380)	0.433 **(0.030)**
1521	CTSW	cathepsin W	0.216 (0.605)	-0.704 **(0.011)**
3576	CXCL8	chemokine (C-X-C motif) ligand 8	0.204 (0.409)	0.406 **(0.038)**
54810	GIPC2	GIPC PDZ domain containing family, member 2	0.167 (0.523)	0.410 **(0.030)**
9698	PUM1	pumilio RNA-binding family member 1	0.160 (0.515)	0.392 **(0.028)**
722	C4BPA	complement component 4 binding protein, alpha	0.109 (0.807)	-0.529 **(0.019)**

91272	BOD1	biorientation of chromosomes in cell division 1	0.104 (0.666)	0.354 **(0.018)**
678	ZFP36L2	ZFP36 ring finger protein-like 2	0.103 (0.510)	0.357 **(0.002)**
8031	NCOA4	nuclear receptor coactivator 4	0.055 (0.805)	0.328 **(0.003)**
966	CD59	CD59 molecule, complement regulatory protein	0.050 (0.760)	0.369 **(1.42E-04)**
1774	DNASE1L1	deoxyribonuclease I-like 1	0.045 (0.768)	0.332 **(2.02E-04)**
5150	PDE7A	phosphodiesterase 7A	0.041 (0.906)	0.425 **(0.003)**
79080	CCDC86	coiled-coil domain containing 86	0.024 (0.974)	0.545 **(0.030)**
1871	E2F3	E2F transcription factor 3	-0.018 (0.958)	0.400 **(0.002)**
197258	FCSK	fucokinase	-0.069 (0.713)	0.385 **(0.001)**
129446	XIRP2	xin actin binding repeat containing 2	-0.085 (0.768)	-0.528 **(0.001)**
7915	ALDH5A1	aldehyde dehydrogenase 5 family, member A1	-0.348 **(0.031)**	-0.094 (0.696)
10422	UBAC1	UBA domain containing 1	-0.378 **(0.017)**	0.048 (0.861)
10193	RNF41	ring finger protein 41, E3 ubiquitin protein ligase	-0.482 **(0.038)**	-0.104 (0.783)

[a]Fold-change between THP-treated and mock-treated samples in unstimulated conditions.

[b]Fold-change between THP-treated and mock-treated samples in PMA-stimulated conditions.

[c]Differential proteins were identified via Log_2 fold change >1.25 and moderated 2-tailed t-test followed by multiple-hypothesis testing correction using the Benjamini-Hochberg procedure with a false discovery rate adjusted *P* < 0.05. Proteins meeting these criteria are indicated by p-values in bold text.

[d]Detected in purified THP by mass spectrometry. See **Supplemental Table 4**.
