# Supplementary material for: Tamm-Horsfall protein augments neutrophil NETosis during urinary tract infection
Source: JCI Insight. 2025 Jan 9;10(1):e180024. doi: 10.1172/jci.insight.180024 (PMC11721310; doi:10.1172/jci.insight.180024)

Full unedited gel for Supplemental Figure 6A.
Lane 1: ladder, Lanes 2-5 purified THP/KO controls, Lanes 6-9: urine input, Lane 10: empty.
Blue lines indicating cropping for image.

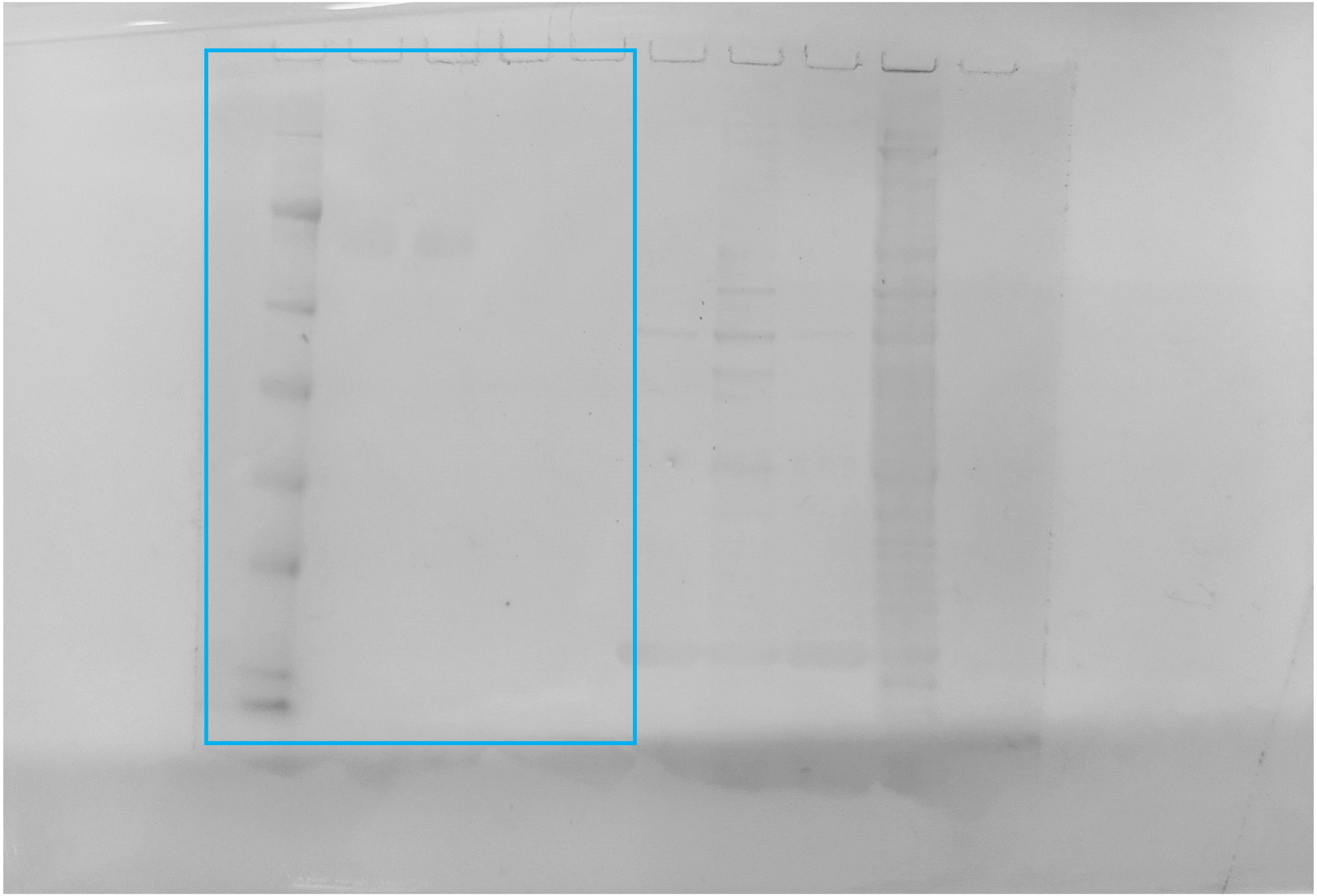

Full unedited gel for Supplemental Figure 6B.
Lane 1: empty,
Lane 2: ladder, Lanes 3-4 purified THP, Lanes 5-10: empty.
Blue lines indicating cropping for image.

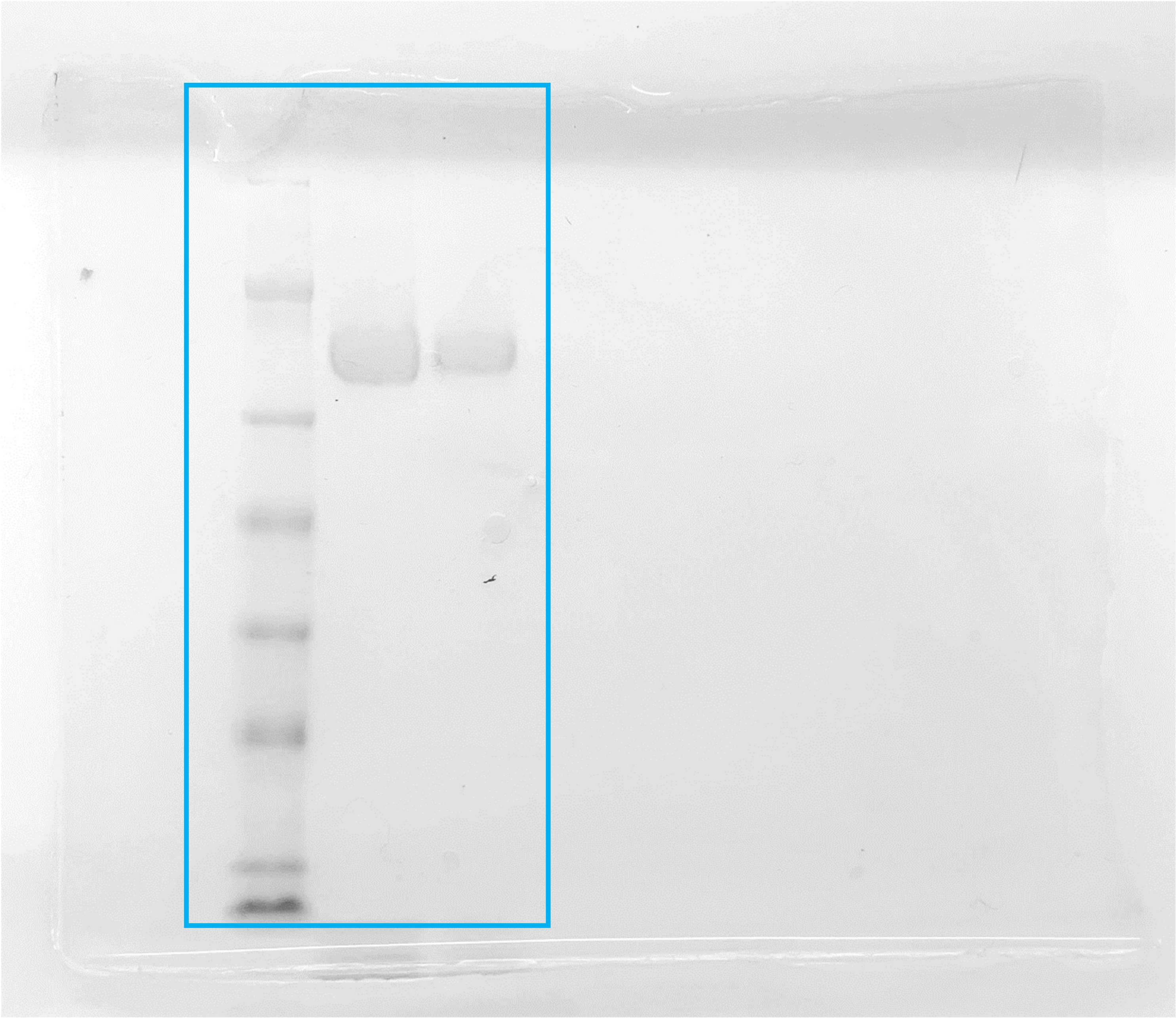

Supplement: Unedited blot and gel images [file jciinsight-10-180024-s200.pdf]
